# Targeting microglia polarization with Chinese herb-derived natural compounds for neuroprotection in ischemic stroke

**DOI:** 10.3389/fcell.2025.1580479

**Published:** 2025-06-10

**Authors:** Lu Yu, Yin Dong, Mincheng Li, Huifang Liu, Cuina Yan, Xiaoxian Li, Yuehua Gu, Liwei Wang, Chuan Xu, Jie Xu, Zhen Yuan, Ming Xia, Jiwei Cheng

**Affiliations:** ^1^ Comprehensive Department of Traditional Chinese Medicine, Department of Neurology, First Department of Integration, Putuo Hospital, Shanghai University of Traditional Chinese Medicine, Shanghai, China; ^2^ Department of Neurology, Shanghai Jinshan Hospital of Integrated Traditional Chinese and Western Medicine, Shanghai, China; ^3^ Department of Traditional Chinese Medicine, Xinqiao Community Health Service Center, Shanghai, China; ^4^ Department of Neurology, Yueyang Hospital of Integrated Traditional Chinese and Western Medicine, Shanghai University of Traditional Chinese Medicine, Shanghai, China

**Keywords:** ischemic stroke, microglia, neuroinflammation, traditional Chinese herbs, natural compounds

## Abstract

Given that ischemic stroke ranks as one of the most fatal diseases globally, it is imperative to develop clinically effective neuroprotective agents for stroke. Microglia serve as innate immune cells for maintaining brain homeostasis, and upon activation, they are well-known to be able to transform into two functional phenotypes, namely, the M1 and M2 types, which can convert each other and exert opposing effects on neurotoxicity and neuroprotection, respectively. Traditional Chinese medicine possesses a deep-rooted and profound history with rich theory in treating cerebrovascular disorders, and its natural compounds have been considered as promising adjunctive therapies. Recently, researchers have been devoting attention to the inflammation-suppressive properties of the compounds from Chinese herbs. These compounds are gradually emerging as adoptable therapeutic agents with wide application prospect for improving stroke outcomes, through regulating microglial polarization to attenuate neuroinflammation. Thereby, we reviewed the functions of microglial cells in inflammation and neuroprotection and explored the regulation of microglial activity by natural compounds to alleviate neuroinflammation and protect neural function after ischemic stroke. Collectively, using natural compounds to suppress the microglia-mediated detrimental inflammatory response, meanwhile enhancing their anti-inflammatory abilities to accelerate neuronal recovery, will be promising therapeutic approaches for ischemic stroke.

## 1 Introduction

Stroke is the second leading cause of death worldwide and its morbidity rates continue escalating (Feigin et al., 2022). Ischemic stroke is the most common type of stroke, accounting for 87% of all cases (Saini et al., 2021), characterized by the formation of arterial thrombosis, which leads to blood flow interruption in a specific brain region, related to the responsible blood vessels (Jadhav et al., 2021), and in turn, triggers a cascade of neurological symptoms (Khoshnam et al., 2017). Currently, reperfusion therapies have been considered practical intervention strategies for ischemic stroke. However, their application remains limited due to the narrow therapeutic time window (Xiong et al., 2018). Thus, crucial problems existing in the management of ischemic stroke lie in discovering effective complementary methods to enhance therapeutic effects and improve stroke outcomes.

Microglia, the immune cells residing in the brain, serve as the initial responders when a cerebral ischemic attack occurs. They are activated within hours and remain active for several days after stroke (Ren et al., 2023). Microglia are renowned for exerting dual roles during the process of cerebral ischemic insult. It can transform from a resting state to an activated state, acquiring beneficial or detrimental bidirectional phenotypes (Ri et al., 2023). In this regard, microglia can regulate the progression of inflammation during brain ischemia by exerting either pro-inflammatory or anti-inflammatory effects (Paul and Candelario-Jalil, 2021; Var et al., 2021). Therefore, different measures aimed at regulating microglial responses to attenuate neuroinflammation can potentially rescue ischemia-damaged neurons.

In recent years, researchers have put emphasis on the redevelopment and utilization of traditional Chinese herbal medicine, which has been extensively applied as complementary and alternative therapies for cerebrovascular diseases under the guidance of theories of traditional Chinese medicine, with remarkable clinical effectiveness and few side effects. Natural compounds, isolated from Chinese herbs, are renowned for their multi-effective and multi-targeting properties (Yu et al., 2020). Emerging evidence has exhibited a broad application prospect of natural compounds in clinic, and the mechanisms of the actions of these compounds on cerebral ischemic injury have gradually been unveiled (Li X. H. et al., 2022; Yu et al., 2022a). The regulatory effects of natural compounds, as well as multi-component extracts, on microglial response have emerged as research hotspots. In this article, we will focus on the neuroprotective actions of natural compounds in cerebral ischemic insult and the regulatory effects of these compounds on microglia-mediated neuroinflammation, along with the related mechanisms involved.

## 2 Mechanisms of microglia in ischemic stroke

### 2.1 Ischemic stroke

Stroke can be classified into two major categories: ischemic and hemorrhagic, in which ischemic stroke represents over 80% of all occurrences (Feigin et al., 2022). In cases of ischemic stroke, circulating thrombi (or atherosclerotic plaques) obstruct cerebral blood vessels, such as the middle cerebral artery (MCA), leading to an interruption in blood and oxygen supply. This disruption results in neuronal necrosis, ultimately damaging brain structure and function (Liu H. W. et al., 2023). Consequently, it leads to a cascade of neurological symptoms, including loss of balance, hemiplegia, decreased sensory and vibratory perception, numbness, reduced or enhanced reflexes, ptosis,and visual field impairment, etc. (Khoshnam et al., 2017). The occurrence of brain ischemia initiates a sequence of detrimental events, including depletion of the ATP-dependent Na+/K+ pump, elevated levels of free cytosolic calcium, overaccumulation of glutamate outside the cell, excessive stimulation of N-methyl-D-aspartatic acid (NMDA) receptor, neuronal excitotoxicity, production of reactive oxygen species (ROS), oxidative stress, mitochondrial dysfunction, and inflammatory response (Jia et al., 2019) (Table 1). These pathological processes interact with each other, causing irreversible damage to neurons, glia and endothelial cells, which further bring about secondary brain injury, manifesting in apoptosis and autophagy/mitophagy in neuronal cells, blood-brain barrier (BBB) damage, hemorrhagic transformation, and vascular brain edema (Khoshnam et al., 2017). As the limited therapeutic time window of stroke, it is imperative to develop efficient complementary intervention methods to improve the clinical effects and the disease outcomes. Neuroinflammation elicited by the excessive activation of microglia is a key pathological process in the brain ischemia. Activated microglial cells are well known to polarize into bidirectional phenotypes, representing proinflammatory or anti-inflammatory action, depending on the specific activation signals they encounter. During the initial stage of brain ischemia, M1 subtype microglia induce neuroinflammation, which causes neuronal death and BBB damage, exacerbating brain ischemic insult. In the late stage of ischemic insult, M2 subtype microglia prompt the process of neuroprotection and neurorestoration, responsible for tissue repair and remodeling. Thus, modulating the equilibrium of microglial polarization to attenuate neuroinflammation might be a promising therapeutic method for treating ischemic stroke.

**TABLE 1:** Different pathological stages of brain ischemia.

Time course	Pathological mechanisms
Acute phase (minutes-hours)	• Decreased cerebral blood flow with inadequate oxygen/glucose delivery • Deprivation of ATP, depolarization of membrane and increased influx of intracellular ion • Accumulation of glutamate, overactivation of AMPA and NMDA • Release of neuromediators (excitotoxicity)
Subacute phase (hours-days)	• Generation of ROS and oxidative stress • Increased expression of cellular adhesion molecules • Activation of microglia and infiltration of leukocyte into the ischemic region • Secretion of pro-inflammatory mediators • Neuronal apoptosis • Autophagy/mitophagy • Dysfunction of BBB and endothelium
Delayed phase (days-weeks)	• Release of trophic factors (BDNF, IGF, GDNF) • Neurogenesis, angiogenesis, axonal remodeling, synaptogenesis • Proliferation of neuronal stem cells

### 2.2 Morphology, structure, and physiological functions of microglia

Microglial cells are derived from myeloid progenitors originating in the yolk sac, and migrate into the brain during their initial stage of development before the blood brain barrier formation (Ginhoux et al., 2010; Waisman et al., 2015). They constitute a highly plastic group of neuroglia, accounting for 5%–15% of the total brain cells, and exhibiting varying proportions across diverse brain regions (Pelvig et al., 2008). Once microglia migrate into the brain, they mature into self-maintaining and renewing populations, without any contribution to peripheral surroundings (Ajami et al., 2007; Ajami et al., 2011). As a kind of resident immune cells, microglia, branched with multiple slender protrusions, constantly patrol, and scan the microenvironment in the brain using their motor branches, interacting with adjacent cells and factors, which is so called resting state of microglia. Evidence suggested that microglia in resting state are not entirely quiescent, but maintain a highly dynamic state (Li D. J. et al., 2018). When stimulated, microglia are rapidly converted into activated state (Cserép et al., 2021). Amoeboid-shaped microglia are observed to be abundant in the ischemic core area between 3 and 7 days in middle cerebral artery occlusion (MCAO) rats (Li Y. et al., 2021). On one hand, activated microglia fulfill their phagocytic function to eliminate pathogens, abnormal proteins, and cellular debris, including apoptotic cells and non-functional synapses, maintaining brain homeostasis (Nguyen et al., 2020). Phagocytic clearance of dead or dying cells by microglia is instrumental for inflammation resolution after stroke (Cai et al., 2019). On the other hand, they generate a huge number of signaling molecules, like pro-inflammatory cytokines, neurotransmitters, and extracellular matrix proteins, for modulating the activities of neurons and synapses (Nguyen et al., 2020). Besides, microglia perform a pivotal role in sustaining blood-brain barrier (BBB)’s integrity. In the early stage of ischemic stroke, microglia secrete pro-inflammatory factors, leading to a disruption in the BBB’s structure and function; in the later stage, they safeguard BBB during neuroinflammation events by releasing anti-inflammatory mediators and engulfing immune cells (Qiu et al., 2021). Furthermore, microglia can stabilize synapses and orchestrate the development of neural circuit by regulating various neural elements, such as astrocytes, myelin and the extracellular matrix (Lukens and Eyo, 2022). However, microglia inadvertently generate ROS during the process of phagocytosis within phagosomes (Cheret et al., 2008). This ROS production can become detrimental in excessive amounts, contributing to oxidative stress, particularly in conditions like stroke (Zhang S. et al., 2024). Collectively, microglia exhibit remarkable phenotypic plasticity in response to destroyed brain homeostasis, such as ischemic condition. These various phenotypes can transform into each other based on changes in microglial morphology or the expression of cell surface antigens.

### 2.3 Activation of microglia after ischemic stroke

Microglia, the initial guardians of immune defense, promptly react to pathological alterations after brain ischemia occurs, maintaining an activated state for several months (Candelario-Jalil et al., 2022). In a transient ischemic stroke rat model, within 24 h after reperfusion, activated microglia become evident in the infarct core, reaching peak levels over a period of 4–7 days. In the peripheral region, microglia were detected to be accumulated within 3.5 h, with a peak at 7 days after reperfusion, which precedes the timing they appeared in the infarct area (Ito et al., 2001). Within the infarct core, microglial activation is initiated by excitotoxic signals that are triggered by brain ischemic insult. In contrast, in the penumbra region, microglial activation is closely tied to innate immune receptors, which are mediated by the release of neuromediators, damage-associated molecular patterns (DAMPs), high-mobility group box-1 (HMGB1) protein and reactive oxygen species (ROS), all originating from ischemia-damaged or -stressed neuronal cells (Khoshnam et al., 2017; Jurcau and Simion, 2022). The disruption of brain homeostasis induced by brain ischemia can trigger the activation of microglia, accompanied by morphological alterations (Ma et al., 2017). Once activated, microglial cells appear proliferated, migrate towards the ischemic-lesion site, and bring about diverse harmful effects, including releasing inflammatory cytokines and cytotoxic substances. They also generate inflammation-suppressive mediators, neurotrophic factors and growth factors that aid in tissue repair and eliminate cellular debris in the late stage of ischemic stroke (Ma et al., 2017). The roles of activated microglia, detrimental or beneficial, largely depend on their phenotypic polarization status after the onset of brain ischemia. Therefore, regulating the equilibrium of microglial phenotypic polarization is recognized as a hopeful therapeutic approach in treating ischemic stroke.

### 2.4 Microglial M1/M2 phenotypical polarization after ischemic stroke

**FIGURE 1:**
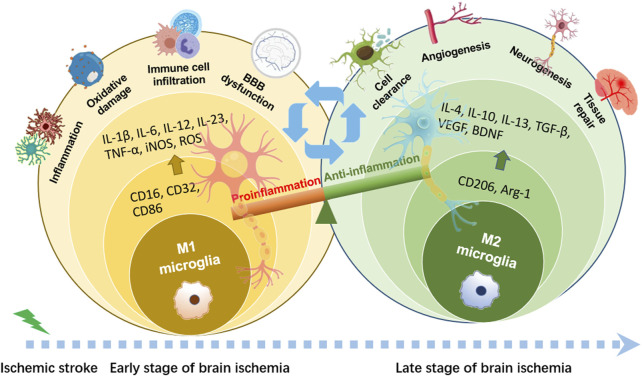
Phenotypic polarization of microglia. Once brain ischemia occurs, microglia rapidly switch from resting state to activated state with amoeboid-like phenotype in morphology. M1 phenotypic microglia mainly release the production of IL-1β, IL-6, IL-12, IL-23, TNF-α, iNOS and ROS, which have cytotoxic effects on neurons, resulting in inflammatory response and oxidative damage. M2 phenotypic microglia mainly secrete the production of IL-4, IL-10, IL-13, TGF-β, VEGF, BDNF, which can attenuate neuroinflammation and promote neurogenesis, for neuronal function recovery and injured tissue repair.

**FIGURE 2:**
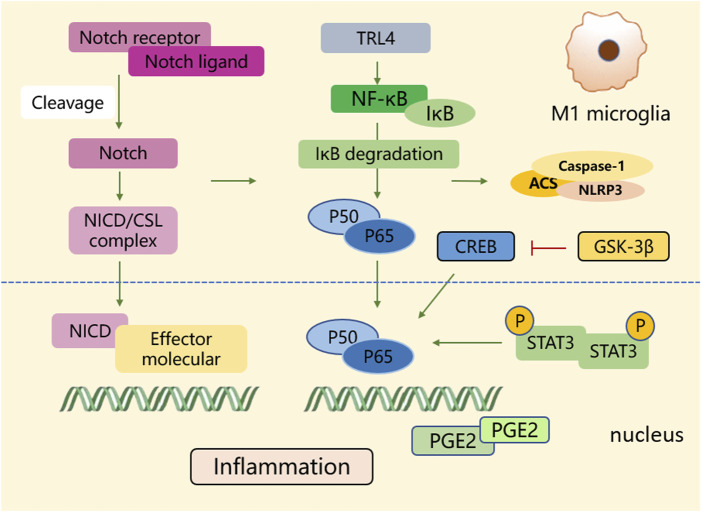
The polarization process of microglial M1 type. During the polarization process of the M1 phenotype, multiple signaling molecules construct a pro-inflammatory network. Signaling pathways such as NF-κB, Notch, STAT3, glycogen synthase kinase-3β, and PGE2 play crucial roles in activating transcriptional genes and downstream signaling cascades.

The “detrimental” M1 type and “beneficial” M2 type are two subtypes of activated microglia, based on their distinct expression profiles of protein and cytokine (Lyu et al., 2021). After the occurrence of ischemia, the release of DAMPs from dead cells triggers microglial activation. Resting microglia are polarized into pro-inflammatory M1 phenotype. In stroke mice with MCAO, M1 microglia was found to secrete inflammatory cytokines, comprising tumor necrosis factor (TNF)-α, interleukin (IL)-1β, IL-6, IL-12, and IL-23, and enhance the levels of inducible nitric oxide synthase (iNOS) and proteolytic enzymes like MMP9 and MMP3 (Zhao et al., 2020; Zhu et al., 2021). M1 phenotype can be identified by detecting specific cell surface markers, like CD16, CD32, and CD86. Various phenotypic states of microglia can be interconverted through their specific activation pathways (Figure 1). In the polarization of the M1 phenotype, multiple signaling molecules interact to form the pro-inflammatory network. TLR4, an important immunorecognition receptor in the neuroinflammation cascade, can be transported to functional areas in the brain. When stimulated by ischemic injury, it recognizes DAMPs, leading to the activation of the p65 subunit of downstream NF-κB pathway, which promotes the transcription of NLRP3 inflammasome components and further regulates inflammatory mediators (Luo et al., 2022; Chen et al., 2023). NLRP3 inflammasome, mainly observed in microglia, plays a crucial role in the inflammatory response following ischemic stroke. Its activation involves the recruitment of pro-caspase-1 to the NLRP3 receptor protein upon ischemia stimuli (Li X. et al., 2024). Suppressing the activation of NLRP3 inflammasome prevents the nuclear translocation of NF-κB p65, modulating microglial polarization and inhibiting microglial apoptosis, thus attenuating neuroinflammation induced by MCAO (Luo et al., 2022; Cai et al., 2024). The nuclear factor kappa B (NF-κB), an upstream signaling for the NLRP3 inflammasome, is capable of activating NLRP3 and inducing M1 microglial polarization (Barnabei et al., 2021; Chen Q. J. et al., 2021). During the acute stage of brain ischemia, the degradation of IκB was found to facilitate the nuclear translocation of NF-κB subunit p65, activating microglia and initiating the transcription of downstream proinflammatory genes (Bi et al., 2024). Notch signaling is activated through the interaction between Notch receptor and ligands, which causes nuclear translocation of intracellular Notch receptor domain (NICD). The NICD then binds to the effector molecules in the nucleus, thus activating the target genes, and subsequently inducing the transcriptional activation of NF-κB and regulating the transformation of microglia into M1 phenotype (Li X. H. et al., 2022). In the MCAO mouse model, the phosphorylation of signaling transducers and activators of transcription (STAT3) was observed to phosphorylate IκB and facilitate NF-κB nuclear translocation, leading to M1-like phenotype transformation (Liu Y. et al., 2024). Meanwhile, glycogen synthase kinase-3β, known as a serine/threonine kinase for controlling NF-κB signaling, dampens the activity of cAMP response element-binding protein (CREB) and increases the accumulation of intranuclear NF-κB induced by brain ischemia (Jover-Mengual et al., 2010). Prostaglandin E2 (PGE2), as a pro-inflammatory mediator, activates downstream signaling pathways through binding to different PGE2 receptors (EP) (Hosoi et al., 2013). Moreover, mTORC1, the contributor to the dysregulation of cellular function following brain ischemia, can mediate M0 microglia to polarize into pro-inflammatory M1 phenotype (Li et al., 2016). These molecules intricately interact with other pro-inflammatory signaling pathways, thus promoting or prolonging the polarization of the M1 phenotype and exacerbating neuroinflammation during the early stage of ischemic stroke (Figure 2).

**FIGURE 3:**
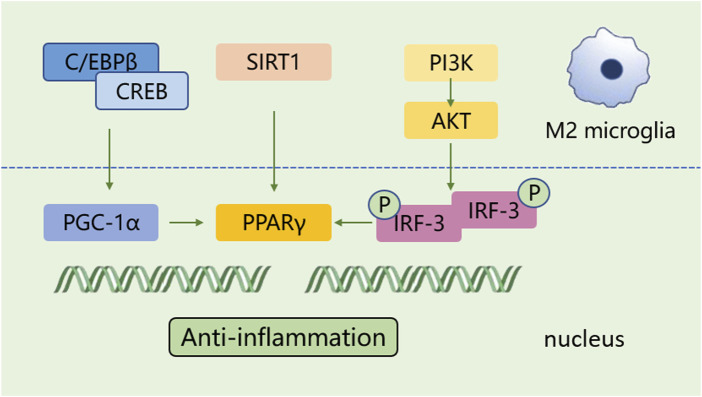
The polarization process of microglial M2 type. The polarization of M2 microglia is regulated by several key signaling molecules, such as PPARγ, CREB, and IRF-3, and others. These molecules control inflammation by regulating gene transcription and establish the functional characteristics of the M2 phenotype.

A shift in microglia phenotype towards an anti-inflammatory M2 state represents a critical repair mechanism during brain ischemia. In contrast to the M1 phenotype, the M2 phenotype generates anti-inflammatory cytokines, such as transforming growth factor (TGF)-β, IL-4, IL-10 and IL-13, as well as increases the levels of growth factors, such as vascular endothelial growth factor (VEGF) and brain-derived neurotrophic factor (BDNF) for neuronal repair at the late stage of ischemic stroke (Qin et al., 2019; Lyu et al., 2021). M2 phenotype with anti-inflammation can be distinguished via some specific biomarkers like CD206 and arginase 1 (Arg1) (Lambertsen et al., 2019; Jurga et al., 2020) (Figure 1). Besides, the activated M2 phenotypes can further be categorized into M2a, M2b and M2c (Almolda et al., 2015). Among that, the M2a subtype is involved in reparative and regenerative processes; the M2b subtype, serving as an intermediate phenotype, is associated with inflammation modulation; M2c subtype participates in neuroprotection, including the clearance of cellular debris and tissue remodeling (Almolda et al., 2015). The polarization of M2 microglia is governed by several key signaling molecules. Peroxisome proliferation-activated receptor γ (PPARγ) serves as a transcription factor to control inflammation. It was found to orchestrate the polarization of microglia and promote the phagocytic ability of microglia, contributing to anti-inflammatory response in mice with tMCAO (Liu X. et al., 2024). Building on this, cAMP response element binding protein (CREB), cooperates with C/EBPβ to promote the expression of PGC-1α, which acts as a transcriptional coactivator to enhance the activity of PPARγ, thus increasing expressions of M2 phenotype-specific genes for maintaining microvascular integrity and ameliorating brain ischemic injury in MCAO rats (Ruffell et al., 2009; Ruan et al., 2019). Additionally, interferon regulatory factor-3 (IRF-3) can be activated through its upstream PI3K/AKT signaling pathway (Tarassishin, et al., 2011), in the form of phosphorylation, facilitating its dimerization and interaction with coactivators. The activated IRF-3 complex translocates into the nucleus and then modulates the transcription of target genes, thereby promoting M2 microglia polarization (Cho et al., 2016; Chistiakov et al., 2017). These signaling pathways collectively establish the functional characteristics of the M2 phenotype during the subacute to chronic phases of brain ischemia (Figure 3).

Notably, some studies pointed out the dichotomous nature of microglia, indicating that they exclusively transform into either M1 or M2 phenotype (Nguyen et al., 2020). However, these views are unable to entirely and accurately capture the intricate physiological characteristics and functions displayed by microglia. Morganti et al. reported that traumatic brain injury elicited a coexistence of different states of microglia, responsible for the production of inflammatory mediators (Morganti et al., 2016). Additionally, microglia were found to occupy a continuous expression spectrum ranging between the M1 and M2 subtypes in ischemia-induced damaged tissue (Hou et al., 2021). Furthermore, with the emergence of single-cell analysis technologies, microglia have been shown to exhibit specific subpopulations under inflammatory conditions that were distinct from neurodegenerative-associated phenotypes, indicating the heterogeneity in activation states of microglia and reflecting their specific functions in relevant environments (Sousa et al., 2018).

Various factors, including severity degrees of ischemic injury, different pathological stages during brain ischemia, the surrounding pathological environment, as well as aging, can influence the polarization of microglia at some extent (Ri et al., 2023). During the initial period of brain ischemia, microglia expressing the M1 phenotype can be detected in the ischemic core area (Subedi and Gaire, 2021; Zhang W. et al., 2021), whereas most microglial cells polarize towards the M2 phenotype in peri-infarct regions. However, a gradual transition towards the M1 phenotype occurs about 1 week after the brain insult, and lasts for weeks thereafter (Zhao et al., 2017; Lyu et al., 2021). The transformation of microglial phenotypes is contingent upon diverse signals they encounter or receive in the pathological environment. Thus, with the appropriate intervention measures, the M1 phenotype might be converted into the so-called protective M2 phenotype, thereby protecting against brain ischemic injury (Ye et al., 2019; Xu et al., 2021). Besides, it has been found that diverse transcriptional mediators closely related to the M1/M2 polarization process represent differential expression patterns within the ischemic region, which serve as action targets for regulating the states of microglia (Holtman et al., 2017). Since M1 and M2 are commonly used to distinguish between distinct microglial phenotypes, the M1 phenotype is typically considered to mediate a pro-inflammatory response that exacerbates ischemic damage, conversely, the M2 phenotype participates in neuronal remodeling and repair processes during the delayed stage of brain ischemia. Therefore, developing therapeutic strategies for ischemic stroke, with the focus on regulating microglial polarization and facilitating their transformation into the neuroprotective M2 phenotype, has become a hotspot that attracts researchers’ attention.

### 2.5 Microglia-mediated neuroinflammation and neuroprotection in ischemic stroke

The inflammatory response is regarded as a pivotal defensive mechanism partially mediated by microglia, tasked with eliminating cellular debris and facilitating tissue repair during the process of brain ischemia (Wei et al., 2020). Once ischemic stroke occurs, resident microglia can first sense and immediately react to danger signals (Lambertsen et al., 2019). They become activated by numerous ischemia-induced damage-associated molecular patterns (DAMPs) (Subedi and Gaire, 2021) and induce a significant inflammatory reaction. The overactivation of microglia generates inflammatory factors, such as IL-6, TNF-α, ROS, and NO, which further drive several types of cell death, including necrosis, apoptosis, and pyroptosis, mediated by the inflammasome and caspases (Xiong et al., 2016). Beyond that, inflammatory factors directly act on mitochondria, leading to abnormalities in their morphology and function. This in turn disrupts mitochondrial dynamics, which are characterized by continuous fusion and fission processes (Gan et al., 2025). The resulting mitochondrial dysfunction activates the NLRP3 inflammasome, promoting microglial M1 polarization. Meanwhile, the shift towards a proinflammatory M1 phenotype accelerates mitochondrial fission. This process leads to the release of damaged mitochondria, ultimately causing neuronal death (Gan et al., 2025). Damaged mitochondria transfer to neurons and fused with neuronal mitochondria, leading to elevated ROS production. The accumulation of ROS and the resultant oxidative stress injury triggers a vicious cycle involving microglial activation, aggregation, and hypersecretion of inflammatory factors (Zhang S. et al., 2024). These factors elicit the recruitment and infiltration of immune cells, including neutrophils, monocytes/macrophages, and T cells, towards the brain parenchyma, leading to a series of inflammatory reactions and subsequent disruption of the BBB, like increased permeability, diminished transport kinetics, and increased vulnerability to toxic or harmful molecules (Ronaldson and Davis, 2020; Zhang S. P. R. et al., 2021). A sequence of stroke-associated adverse outcomes is then followed, including vascular brain edema, hemorrhage transformation, and leakage of toxic substances from the BBB (Ma et al., 2017; Yang et al., 2019). Thereby, blocking the production of pro-inflammatory mediators from microglia to attenuate BBB disruption and tissue damage represents a hopeful therapeutic method for ischemic stroke (Lu et al., 2019; Liu et al., 2020b).

In contrast to M1 microglia, activated M2 microglial cells exhibit neuroprotective and neurorestorative functions during the delayed stage of brain ischemia (Miron et al., 2013). On one hand, M2 microglia produces numerous cytokines, releases various growth factors, and generates several neurotrophic factors, which provide significant assistance in suppressing inflammation, protecting neurons, and promoting tissue repair following ischemic insult (Wang Y. et al., 2022). For another, M2 microglia phagocytize cell debris and myelin fragments and initiate the processes of synaptogenesis and neurogenesis, contributing to mitigating the harmful events and promoting tissue repair (Jia et al., 2021). Additionally, they phagocytize immune cells within damaged brain tissue when encountering special “eat me” signals emitted by endangered cells, thereby modulating inflammatory response (Yu F. et al., 2022). Moreover, M2 microglia promote the restoration of neuronal functions, encompassing neurogenesis, axonal regeneration, angiogenesis, oligodendrocyte production and remyelination, healing the injured tissue (Hu et al., 2015; Zhang S. P. R. et al., 2021). Beyond that, M2 microglia enhance the proliferation, differentiation, survival, and integration of neural progenitor cells (NPCs) in the ischemia-damaged brain (Deierborg et al., 2010). Elevating the number of insulin-like growth factor-1 (IGF-1)-expressed microglial cells following stroke can attenuate cellular apoptosis and facilitate the proliferation and differentiation of neural stem cells (NSCs) (Thored et al., 2009). Amplifying microglial reparative capabilities improves oligodendrocyte regeneration and remyelination during the late stage of ischemia (Shi et al., 2021). Moreover, microglia can stimulate vessel growth and angiogenesis directly or indirectly, representing their improvement effects on blood vessel reconstruction after stroke (Lyu et al., 2021). Apart from that, the polarization of M2 microglia enhances neural regeneration, leading to sustained neuroprotectiveness in the chronic phase of cerebral ischemia (Jin et al., 2014; Zhu J. et al., 2019; Shang et al., 2020).

Though detailed molecular mechanisms underlying the neuroprotective properties of microglial cells remain incompletely understood, numerous evidence supports their beneficial effects on neuronal recovery post stroke (Zhang W. et al., 2021). Altogether, regulating the activation and phenotypic transformation of microglia, to suppress M1-induced neuroinflammation and promote M2-associated neuronal recovery and tissue repair offers promising intervention strategies for ischemic stroke.

## 3 Regulatory effects of natural compounds from Chinese herbs on microglial response in ischemic stroke

Traditional Chinese herbal medicine has experienced a profound history in treating different diseases, including ischemic stroke. They have been demonstrated to possess remarkable efficacy with few side effects through extensive long-term clinical practices, such as honghua injection (Li L. et al., 2022), danhong injection (Wang et al., 2016), shuxuetong injection (Fang et al., 2020), and xuesaitong injection (Feng et al., 2021). Diverse natural compounds isolated from these Chinese herbs exhibit a wide range of pharmacological functions (Supplementary Table S1). Hence, it is necessary to elucidate the related mechanisms involved in pharmacological properties of the compounds. Substantial evidence indicates that natural compounds can effectively attenuate brain ischemic damage, foster neuronal recovery and improve prognosis by modulating the polarization of microglia. They regulate the signaling pathways and molecular targets to inhibit M1 microglial polarization and prompt M2 phenotypic transformation, thereby ameliorating inflammatory responses, maintaining BBB function, inhibiting neuronal apoptosis, attenuating oxidative stress, relieving neuronal excitoxicity, and promoting neurogenesis and angiogenesis.

Since activated microglia express mixed M1 and M2 markers with varying degrees in the damaged tissue, the modulation effects of natural compounds on microglia merits further reconsideration. It is rational to believe that the compounds possibly influence the equilibrium of microglial polarization, rather than directly induce exclusive activation of either M1 or M2 subtype (Ri et al., 2023). Herein, we will focus on various regulators engaged in the microglial response following cerebral ischemic injury, summarize their molecular mechanisms, and explain how representative natural compounds attenuate microglia-mediated neuroinflammation through these mechanisms.

### 3.1 Flavonoids

#### 3.1.1 Wogonin

Wogonin is the main active constituent separated from *Scutellaria baicalensis* Georgi (Zhao et al., 2019). It has been demonstrated to possess an extensive spectrum of pharmacological actions, like anti-inflammation and anti-oxidation. Yeh et al. reported that wogonin could suppress the generation of PGE2 and nitric oxide (NO) in lipopolysaccharide (LPS)/interferon (IFN) γ-induced BV2 microglial cells through the modulation of the Src-MEK1/2-(ERK)1/2-NFκB signaling pathway, which was responsible for the alleviation effect of wogonin on neuroinflammation (Yeh et al., 2014).

#### 3.1.2 Ginkgetin

Ginkgetin is a flavonoid dimer extracted from ginkgo, exhibiting anti-cancer, anti-inflammatory, anti-microbial, anti-adipogenic, and neuroprotective activities (Adnan et al., 2020). In recent years, it has been gradually discovered in more than 20 different plant species, most of which are well-known for their use in traditional medicine (Cankaya et al., 2023). PPARγ is a ligand-responsive nuclear transcription factor and has been identified to participate in various pathological processes, including regulating the transformation of microglia/macrophage to resolve inflammation and promote brain repair (Fang et al., 2024). Tang et al. reported that ginkgetin treatment shifted microglia from M1 towards M2 subtype, inhibited neuroinflammation, and exerted neuroprotective effects in OGD cellular model and in MCAO rats. However, the ginkgetin’s effects were abolished by PPARγ antagonist GW9662, indicating that the promotion effect of ginkgetin on M2 microglial polarization was mediated through PPARγ signaling pathway (Tang T. et al., 2022).

#### 3.1.3 Baicalin

Baicalin, a pleiotropic flavonoid ingredient, has attracted considerable interest for its neuroprotective effects on kinds of inflammatory and demyelinating diseases in central nervous system (Li Y. et al., 2020; Ai et al., 2022). Baicalin has been found to regulate the activation of microglia and astrocytes in the hippocampus of LPS-treated mice, resulting in neuroinflammation attenuation (Shah et al., 2020). Consistently, *in vitro* experiments using LPS-induced BV-2 microglial cells revealed that baicalin reduced the production of inflammatory mediators. Importantly, the inflammatory effects of baicalin were realized by blocking toll-like receptor 4 (TLR4)-mediated signaling transduction through TLR4/MyD88/NF-κB and mitogen-activated protein kinases (MAPK) pathways (Li B. et al., 2022). In response to LPS-induced neuroinflammation in mice and microglial cell line, baicalin was evidenced to decrease microglia-mediated inflammation via downregulating HMGB1 level in a Sirtuin 1 (SIRT1)-dependent manner (Li Y. et al., 2020). These studies demonstrate that baicalin modulates microglia activation in LPS-induced neuroinflammatory models. Moreover, Xiao et al. set up a chronic cerebral hypoperfusion animal model and found that baicalin modified microglia polarization towards an anti-inflammatory phenotype and inhibited pro-inflammatory cytokines production (Xiao et al., 2023). In MCAO mice and OGD/R-induced BV2 cells, Wang et al. observed that baicalin inhibited microglia activation by upregulating the level of TREM2, thereby suppressing inflammatory responses (Wang H. et al., 2024).

#### 3.1.4 Icariin

Icariin is a flavonoid constituent extracted from Chinese medicinal herb, Epimedium, which is commonly used to treat bone fracture and bone loss for thousands of years (Wang et al., 2018). Recently, icariin has captured more attention due to its multiple pharmacological properties, like anti-aging, anti-oxidation, and anti-inflammation (Liu et al., 2015). Current studies have validated neuroprotective actions of icariin against neurodegenerative diseases (Zheng et al., 2019). Nuclear factor erythroid 2 related factor 2 (Nrf2) serves as a key facilitator of endogenous inducible defense mechanisms, encoding diverse array of enzymes with anti-oxidative activities. Activated Nrf2 possesses the properties of anti-oxidation and anti-inflammation (Zheng et al., 2019). Zheng et al. pointed out that icariin modulated microglial polarization, effectively mitigating LPS-induced pro-inflammatory factors in microglia. Furthermore, the triggering of Nrf2 signaling pathway was evidenced to engage in icariin-mediated anti-inflammatory effects (Zheng et al., 2019). Additionally, in oxygen-glucose deprivation/reoxygenation (OGD/R)-damaged microglial cells, icariin downregulated the levels of IL-1β, IL-6 and TNF-α by the suppression of IRE1α-XBP1 signaling pathway, implying that its anti-inflammation effects could be achieved by mitigating endoplasmic reticulum stress (Mo et al., 2021).

#### 3.1.5 Quercetin

Quercetin, existing in diverse traditional Chinese medicinal herbs, tea, fruits, and vegetables, is a common plant flavonoid with kinds of pharmacological effects, like anti-fibrosis, anti-virus, anti-cancer, anti-inflammation, as well as anti-oxidation (Russo et al., 2012). Quercetin has already been approved for clinical use owing to its suppressive effect on the activity of tyrosine kinase (Han et al., 2021). Han et al. reported that quercetin could regulate the LPS-induced proliferation and phagocytosis of primary microglia. Moreover, it suppressed inflammatory response in LPS-treated BV2 microglial cells without compromising their cellular viability. This suppression was likely attributed to its ability in reducing the expressions of NLR family, pyrin domain-containing 3 (NLRP3) inflammasome and pyroptosis-related proteins by promoting mitophagy (Han et al., 2021).

#### 3.1.6 Hydroxysafflow yellow A

Hydroxysafflow yellow A (HSYA), a main bioactive compound extracted from *Carthami flos*, has been extensively used for cardio- and cerebrovascular diseases with its diverse biological functions, such as anti-oxidation, anti-inflammation, and anti-apoptosis, etc. (Yu et al., 2022b). It was reported that HSYA activated TLR9 in microglial cells of the ischemic cortex in rats with MCAO, and then blocked the pro-inflammatory NF-κB pathway from day 1 to day 7. However, its inflammation-suppressive action was abolished when silencing TLR9 in OGD/R-exposed primary microglial cells, indicating that the anti-inflammation effect of HSYA was tightly linked to reprograming the TLR9 signaling pathway (Gong et al., 2018). Similarly, in a Transwell co-culture system comprising microglia and neurons, HSYA treatment could suppress TLR4 expression in the LPS-activated microglia, resulting in reducing neuronal damage (Lv et al., 2016).

#### 3.1.7 Schaftoside

Schaftoside exists in fruits, vegetables, nuts, seeds, herbs, spices, stems, flowers, as well as in tea and red wine (Romano et al., 2013). Zhou et al. reported that schaftoside could inhibit the generation of inflammation-promoting cytokines, like IL-1β, TNF-α and IL-6 in OGD/R-injured BV2 microglial cells through suppressing the activity of TLR4/MyD88 signaling pathway (Zhou et al., 2019). Mitochondria are critical organelles within microglia that regulate their functions. Mitochondrial dynamics, the balance between mitochondrial fission and fusion, are involved in numerous cellular pathways, including inflammation and apoptosis (Tabara et al., 2025). Dynamin-related protein 1 (Drp1) is a major modulator of mitochondrial fission, phosphorylation of Drp1 at the Ser616 site can speed up the fission of mitochondria (van der Bliek et al., 2013). Furthermore, schaftoside has been evidenced to inhibit expression level, phosphorylation, and translocation of Drp1 in OGD-conditioned BV2 microglial cells, hindering the fission of mitochondria and thereby counteracting neuroinflammatory response (Zhou et al., 2019).

### 3.2 Polyphenols

#### 3.2.1 Gastrodin

Gastrodin, an effective polyphenol isolated from *Gastrodia elata*, possesses diverse neuroprotective effects, including attenuating brain ischemic damage (Zeng et al., 2006), ameliorating cytotoxicity mediated by hypoxia in cortex neurons (Xu et al., 2007), and safeguarding hippocampal neurons from neurotoxicity elicited by Aβ peptide (Zhao et al., 2012). Yao et al. pointed out that gastrodin could not only regulate the activation and the population size of microglia, but also suppress the LPS-induced inflammatory factors in both BV2 and primary microglial cells, as well as in three-day-old rats (Yao et al., 2019). Further study revealed that gastrodin inhibited inflammation and cell proliferation mainly through regulating the Wnt/β-catenin pathway (Yao et al., 2019). Interestingly, gastrodin was observed to hinder the release of pro-inflammatory mediators and concomitantly promoting the secretion of neurotrophic factors in OGD-stimulated BV2 microglia (Lv et al., 2021). The dual roles gastrodin performed in microglia might be due to its regulatory ability on MAPK signaling pathway (Lv et al., 2021). Additionally, gastrodin exerted neuroprotective effects against the hypoxic-ischemia brain damage through the suppression of pro-inflammation mediators in activated microglia by the renin-angiotensin (RAS) system and the SIRT3 pathway (Liu S. J. et al., 2018). Moreover, it was found to block the migration of activated microglial cells through the Notch-1 pathway in LPS-stimulated BV2 microglia and postnatal rats (Yao et al., 2022). Most of these research findings have been observed in cellular models, indicating the inhibitory effects of gastrodin on microglial activation, proliferation, migration, and associated inflammation; however, validation in stroke animal models is still lacking.

#### 3.2.2 Curcumin

Curcumin, a hydrophobic polyphenol separated from *Curcuma longa*, possesses a wide spectrum of therapeutic benefits, including inflammation-suppressive and antioxidative effects. Extensive studies have elucidated that curcumin could dampen the generation of reactive oxygen species and subsequently alleviate neuroinflammatory injury (Patel et al., 2020). Liu et al. reported that curcumin exhibited remarkable regulatory effects on microglia, facilitating M2 microglial polarization and inhibiting microglia-mediated inflammatory responses in mice subjected to dMCAO (Liu Z. et al., 2017). Similarly, curcumin was found to alleviate white matter lesions and reduce brain tissue loss at 21 days post stroke in MCAO mice. Cellular experiments further validated that curcumin could attenuate microglial pyroptosis induced by LPS and ATP, which is considered as a type of inflammatory programmed cell death (Ran et al., 2021). Emerging biomaterial-integrated drug delivery systems are employed to enhance the efficacy of natural compounds for ischemic stroke treatment, particularly focusing on modulating microglial inflammatory responses. Wang et al. prepared nanoparticles (NPs) using a single-emulsion method and encapsulated curcumin in mPEG-b-PLA block copolymer NPs to assess the intervention effects of NPcurcumin on brain ischemic insult (Wang Y. et al., 2019). The findings indicated that NPcurcumin was more efficient compared to curcumin alone in maintaining the integrity of BBB, dampening the activation of M1 microglia, and reducing the levels of inflammatory factors (Wang Y. et al., 2019). Moreover, for targeting the stroke cavity and ensuring sustained on-site drug release, Zhang et al. synthesized a curcumin-loaded injectable hydrogel with double ROS-scavenging effect. They found that curcumin loaded into hydrogels with enhanced ROS-scavenging capacity could facilitate anti-inflammatory microglia polarization through hindering the translocation of p47-*phox* and p67-*phox*, and subsequently enhancing neuroplasticity (Zhang S. et al., 2024).

#### 3.2.3 Resveratrol

Resveratrol, a natural polyphenolic compound presenting in grapes, peanuts, plums, red wines, as well as other dietary sources, exhibits multiple biological activities, including anti-oxidation, anti-inflammation, anti-cancer, and neuroprotection (Zhang L. X. et al., 2021). Recently, resveratrol has been found to facilitate M2 microglia polarization for neuronal restoration following cerebral ischemia (Yang et al., 2017). Decreased expression of astroglial type-1 glutamate transporter (GLT-1) in the hippocampus after stroke leads to an increase in glutamate levels, which is considered as a key facilitator in neurotoxicity (Girbovan and Plamondon, 2015). Girbovan et al. reported that resveratrol could reverse the global ischemia-induced downregulation of GLT-1 level and inhibit the overexpression of CD11b/c and glial fibrillary acidic protein (GFAP), suggesting beneficial roles resveratrol performed in regulating microglial activation and attenuating excitotoxic cascade (Girbovan and Plamondon, 2015). Nrf2 is coupled with its cytoplasmic inhibitor, kelch-like ECH-associated protein 1 (KEAP1), functioning as an intracellular safeguard against oxidative insults (Chen S. et al., 2021). In the case of oxidative stress, Nrf2 dissociates from its inhibitor KEAP1, translocates towards the nucleus and interacts with the antioxidant response element, enhancing the activities of antioxidant enzymes (Kobayashi and Yamamoto, 2005). Since miR-450b-5p serves as a potential therapy target in inflammatory disorders (Luo et al., 2019), Liu et al. revealed that resveratrol could elevate Nrf2 level by modulating miR-450b-5p/KEAP1 axis, leading to the promotion of M2 microglial polarization, thus exerting neuroprotective effects against ischemic injury in MCAO rats (Liu J. et al., 2023).

The neuroprotective or neurotoxic roles NF-κB performed depends on biological functions of the subunits which compose the transcription factor. RelA (p65) and p50 are the subunits of NF-κB family, and p50/RelA complexes can be induced by neurotoxic stimuli (Inta et al., 2006). Since acetylation of RelA at the K310 site affects the function of p50/RelA complexes, Mota et al. combined class I histone deacetylase inhibitors (HDACi) MS-275 (20 μg/kg) with resveratrol (680 μg/kg) at low doses and found that the combination reduced infarct volume and neurological deficits in dMCAO mice, hindered the binding ability of RelA to the Nos2 promoter. Consequently, the combination reduced the levels of Nos2, IL-6, IL-1β, avoided leukocyte infiltration in the ischemic area, blunted the activation of microglia/macrophages, and weakened the immunoreactivity of iNOS and CD68 in Iba1-positive cells (Mota et al., 2020). All evidence suggested that the combination of MS-175 and resveratrol exerted anti-inflammation effects through directly inhibiting microglia/macrophage activation, achieving greater efficacy than either drug alone, even when the individual drugs were used at 100-fold higher doses.

#### 3.2.4 6-shogaol

*Zingiber officinale* Roscoe (ginger), a Chinese medicinal herb, has long been applied for headaches, colds, nausea, and emesis, etc. (Mao et al., 2019). Many bioactive components containing in ginger have been identified, among which 6-shogaol is a pungent phenolic component with remarkable pharmacological properties (Mao et al., 2019). Han et al. reported that 6-shogaol elevated PPAR-γ level and subsequently reversed the enhanced activity of NF-κB to block the release of inflammatory mediators in LPS-treated BV2 microglial cells (Han et al., 2017). Gaire et al. reported consistent findings, which indicated that 6-shogaol could attenuate microglia-mediated neuroinflammation, and their animal study in MCAO mice further validated this effect (Gaire et al., 2015).

#### 3.2.5 Paeonol

Paeonol, a major polyphenolic ingredient from *Paeonia Iactiflora* Pall., possesses various pharmacological properties, like anti-inflammation, anti-tumor, and neuroprotection (Zhang et al., 2019). Paeonal exerted anti-inflammation effects in LPS-activated N9 microglia cells, mainly via suppressing the TLR4 signaling pathway (He et al., 2016). Moreover, paeonol was observed to inhibit inflammatory responses stimulated by LPS/IFN-γ and reduced ATP-induced enhanced migratory activity in BV2 microglia, which was attributed to its ability in modulating AMPK/GSK3 pathway. Improved rotarod performance and decreased microglial activation were observed in mice with systemic inflammation induced by LPS (Lin et al., 2015). However, studies on the regulation of microglia by paeonol in stroke animal models remain limited.

### 3.3 Terpenes

#### 3.3.1 Triptolide

Triptolide, a kind of epoxidized diterpene lactone extracted from *Triprerygium*, exhibits favorable bioactivities in cancers and inflammatory and autoimmune disorders (Chen J. et al., 2022). Zhang et al. reported that triptolide held the ability to repress the synthesis of NO and iNOS in LPS-treated microglial cells and protected neuronal cells from microglia-mediated inflammation. Further, EP2/protein kinase A (PKA) pathway was evidenced to be a major contributor to suppressive effects of triptolide on NO production in microglia (Zhang et al., 2015). Zhou et al. found that triptolide could suppress the polarization of M1 microglia by modulating the CTSS/Fractalkine/CX3CR1 pathway, additionally, attenuate HT-22 cell apoptosis via crosstalk with BV-2 microglial cells (Zhou et al., 2024). Ki20227, a specific blocker of colony-stimulating factor 1 receptor (CSF1R), is responsible for modulating inflammatory response and neuronal synaptic plasticity (Jiang et al., 2022). Du et al. combined triptolide with Ki20227 to evaluate the neuroprotective action of this combination in mice with ischemic stroke. The combination was exhibited to upregulate the expression of synaptic proteins, improve the density of dendritic spines, especially, downregulate the expression of microglial marker Iba1 in stroke mice, which were achieved by inhibiting CSF1R signal and triggering BDNF-Akt and autophagy pathways (Du et al., 2020).

#### 3.3.2 Ilexonin A

Ilexonin A, a pentacyclic triterpene existing in the medicinal herb *Ilex pubescens*, exhibits marked effects in cardiovascular disease, angina, and vasculitis (Luo et al., 1995). Ilexonin A promotes blood circulation for therapeutic actions through its anti-thrombotic and inflammation-suppressive properties (Xu et al., 2016). Xu et al. observed that ilexonin A elevated the number of GFAP-expressed astrocytes in the peri-infarct region after MCAO-induced ischemic injury at 1, 3, and 7 days. However, at 14 days, the number of these cells was decreased compared to the ischemia group. Besides, ilexonin A lowered the numbers of Iba-1 positive microglial cells at each time point (Xu et al., 2016). Another similar study suggested that the numbers of astrocytes in the hippocampal CA1 area promptly increased following ischemic stroke onset, and this augmentation was further amplified after ilexonin A treatment (Xu A. L. et al., 2020). On the other hand, microglial cells remained inactive after ischemia, but was observed to be activated following ilexonin A treatment (Xu A. L. et al., 2020), that were inconsistent with the previously mentioned findings. The reason behind the discrepancy may be that microglia in the CA1 area of hippocampus have not been immediately activated after the occurrence of ischemic insult, whereas those in the peri-infarction region have already been rapidly activated. The observation indicated that different injury regions can lead to varying degrees of activation and proliferation of astrocytes and microglia, and ilexonin A acted as a neuroprotective agent through regulating activities of astrocytes and microglia for attenuating inflammatory responses (Xu A. L. et al., 2020).

#### 3.3.3 Artesunate

Artesunate is derived semi-synthetically from artemisinin and has anti-inflammatory properties. Okorji et al. found that artesunate could reverse the elevated levels of PGE2 stimulated by LPS + IFNγ in BV2 microglia, which was mediated by reduction in COX-2 and mPGES-1. Besides, it decreased the levels of inflammatory cytokines in activated BV2 microglial cells and its suppressive effects were obtained by interfering with p38 MAPK and NF-κB signaling (Okorji and Olajide, 2014). Further, Liu et al. reported artesunate’s anti-inflammatory effects in mice subjected to distal middle cerebral artery occlusion (dMCAO). Artesunate could ameliorate inflammatory responses by reducing neutrophil infiltration, suppressing microglial activation, quenching the secretion of inflammatory cytokines, and restraining the triggering of the NF-κB signaling (Liu Y. et al., 2021).

### 3.4 Alkaloids

#### 3.4.1 Berberine

Berberine is a bioactive isoquinoline alkaloid with extensive pharmacological properties in several central nervous system (CNS) disorders, such as ischemic stroke, Alzheimer’s disease and Parkinson’s disease (Lin and Zhang, 2018). Recent years, researchers put the hotspot on manipulating the peripheral environment or related factors to regulate microglia functions, rather than directly targeting microglia and neuroinflammation (Ni et al., 2022). Ni et al. investigated the contribution of gut-brain axis signals in the berberine-regulated microglia polarization after cerebral ischemia and found that berberine regulated the transformation of microglia and ameliorated inflammatory response in a microbiota-dependent manner. Importantly, the transmission of gut-brain axis signals mediated by berberine was mainly due to the stimulation of intestinal H_2_S on vagal nerve activity, through the transient receptor potential vanilloid 1 (TRPV1) receptor (Ni et al., 2022). Additionally, berberine was shown to inhibit microglia polarization towards the M1 subtype and promote their shift towards the M2 subtype in mice subjected to tMCAO. These effects were validated to occur through AMP-activated protein kinase (AMPK)-dependent mechanisms (Zhu J. et al., 2019). Furthermore, Kim et al. suggested that berberine diminished global ischemia-induced cellular apoptosis by inhibiting the reactive astrogliosis and microglia activation via triggering the PI3K/Akt pathway (Kim et al., 2014).

#### 3.4.2 Tetramethylpyrazine

Tetramethylpyrazine (TMP) is the major bioactive alkaloid separated from *Ligusticum chuanxiong* Hort (Lin et al., 2022) and commonly used in the treatment of cardiovascular, nervous, and digestive system conditions with its extensive physiological functions, including anti-oxidation, anti-inflammation, anti-apoptosis, angiogenesis regulation, and endothelial protection, etc., (Lin et al., 2022). TMP has been reported to inhibit the LPS-induced overproduction of NO and iNOS in N9 microglial cells through restraining the activity of MAPK and PI3K/Akt signaling pathway (Liu et al., 2010). In a rat model of permanent cerebral ischemia, TMP was shown to decrease the percentage of activated macrophages and microglia and ameliorate pro-inflammatory responses after brain ischemia. Further, targeting macrophages/microglia by stimulating Nrf2/HO-1 pathway actively contributed to TMP-mediated neuroprotection (Kao et al., 2013). Moreover, TMP was observed to prevent demyelination and promote remyelination in rats with MCAO based on MRI-diffusion tensor imaging (DTI) and histopathology, and was further discovered to prompt the transformation of microglia towards M2 phenotype, acting through JAK2/STAT1/2 and GSK3-NFκB pathways (Feng et al., 2023).

### 3.5 Glycosides

#### 3.5.1 Astragaloside IV

Astragaloside IV (AS-IV) is a cycloartane-type triterpene glycoside compound separated from Chinese herb *Astragalus mongholicus* Bunge (Zhang et al., 2020). AS-IV has been reported to attenuate behavioral and neurochemical deficits due to its antioxidant, anti-apoptotic, and anti-inflammatory properties in Alzheimer’s disease, Parkinson’s disease, cerebral ischemia, and autoimmune encephalomyelitis; additionally, it serves as a neuroprotector by reducing spontaneous neuronal excitability (Zhang et al., 2020). Li et al. suggested that AS-IV facilitated the shift of microglia/macrophage towards M2 subtype in a PPARγ-dependent manner, which contributed to enhancing neurogenesis, angiogenesis, and neurological functional recovery in rats with tMCAO (Li L. et al., 2021). Gao et al. constructed the molecular regulatory network of lncRNA/miRNA/mRNA to form the pyroptosis-associated competitive endogenous RNA (ceRNA) regulatory relationship specifically for NLRP3 molecules, and LOC10255978/miR-3584-5p/NLRP3 was included. In MCAO rats and OGD/D-treated primary rat microglial cells, AS-IV was found to inhibit microglia inflammatory reaction and pyroptosis by downregulating NLRP3 through LOC10255978, thereby exerting neuroprotective effects (Gao et al., 2024). Additionally, AS-IV was observed to suppress the activation of microglia and alleviate the secretion of inflammatory mediators through containing TLR4 signaling pathway and NLRP3 inflammasome overactivation, thereby restoring cognitive impairment in mice with bilateral common carotid artery occlusion (Li et al., 2017). Besides, AS-IV was displayed to decrease the levels of inflammatory mediators in BV2 and primary microglial cells, mainly mediated by stimulating nuclear factor erythropoietin-2-related factor 2 (Nrf2)/heme oxygenase-1 (HO-1) via the ERK pathway (Li C. et al., 2018). Moreover, it was reported to promote microglial polarization from M1 to M2 subtype in AMPK-dependent metabolic pathways after ischemic stroke (Li et al., 2024a). These findings were reciprocally validated through animal and cellular experimental models.

#### 3.5.2 Cycloastragenol

Cycloastragenol (CAG), an activated derivative of astragaloside IV, is the hydrolysis product of astragaloside IV (Zhou et al., 2012), with pharmacological effects of activating telomerase and anti-aging (Yu et al., 2018). Chen et al. reported that CAG promoted M2 microglia and suppressed M1 polarization by activating Nrf2 signaling pathway and inhibiting NF-κB in LPS-stimulated BV-2 cells and ischemic mouse brain (Chen T. et al., 2022). In addition, it reduced the levels of pro-inflammatory cytokines and restrained the activation of microglia and astrocytes in ischemic brain, which was attributed to its actions on regulating SIRT1 expression, blunting p53 acetylation and inhibiting NF-κB activation (Li M. et al., 2020).

#### 3.5.3 Salidroside

Salidroside, a phenylpropanoid glycoside separated from the root of *Rhodiola rasea* L. has various therapeutic effects in aging, cancer, inflammation, oxidative stress, and kinds of neurological disorders, like stroke and Alzheimer’s disease (Magani et al., 2020). Using network pharmacology, transcriptome sequencing, macromolecular docking and molecular biology techniques, Zhang et al. revealed that salidroside inhibited the activation of microglia by inducing GSK3β phosphorylation, and in turn targeting downstream Nrf-2, facilitating β-catenin accumulation, and ultimately exerted protective effects against hypobaric hypoxia-induced brain injury (Zhang X. et al., 2024). Besides, Fan et al. simulated the hypoxic microenvironment in BV2 microglia, and investigated the change of cell metabolites using a cell microfluidic chip-mass spectrometry (CM-MS) system. The findings showed that microglial hypoxic inflammation was associated with cell energy metabolism, in which the process of metabolism changed from oxidative phosphorylation to glycolysis, and salidroside could reverse this change to further alleviate microglial hypoxic inflammatory injury (Fan et al., 2022). Although these studies did not employ stroke models directly, the hypoxia model shares numerous overlapping mechanisms with ischemic stroke, including energy depletion, BBB disruption, and neuroinflammation, thus allowing for cross-validating the underlying pathological pathways of ischemic stroke. Besides, salidroside was found to facilitate the polarization of M2 macrophage/microglia following ischemic injury, and initiate a shift from M1 towards M2 subtype in primary microglial cells. Moreover, it enhanced microglia phagocytic activity and attenuated microglia-mediated inflammatory cytokine release. When oligodendrocytes were cocultured with salidroside-treated M1 microglia, a marked acceleration in differentiation of oligodendrocyte was observed (Liu X. et al., 2018). Similarly, salidroside was found to block inflammatory responses in MCAO rats through the regulation of TLR4/NF-κB pathway (Liu J. et al., 2021) and PI3K/Akt pathway (Wei et al., 2017).

#### 3.5.4 Ginsenoside Rd

*Panax ginseng* and *Panax notoginseng*, included in the Araliaceae family, are commonly utilized in clinical practice as functional herbs. They both contain crucial bioactive ingredients, like ginsenosides, which exhibit numerous pharmacological effects on the nervous system (Liu H. et al., 2020). Ginsenoside Rd, a kind of monomer, separated from these two traditional Chinese herbs (Tang K. et al., 2022), has long been applied in treating ischemic stroke with remarkable efficacies and few adverse reactions (Zhang G. et al., 2016). It has been reported to improve the outcome of ischemic stroke patients, and the therapeutic effect may result from its capacity of suppressing the activity of proteasome in microglia, and sequential inflammatory responses (Zhang G. et al., 2016).

#### 3.5.5 Ginsenoside Rb1

Ginsenoside Rb1 stands as another important active ingredient within ginsenosides, the bioactive saponins from *Panax ginseng* or *Panax notoginseng* (Liu H. et al., 2020). In recent years, more attention has been drawn for its remarkable properties in the nervous system (Gong et al., 2022). It could decrease the level of Iba1 and suppress microglial activation, thus attenuating neuroinflammation in mice with systemic LPS treatment (Lee et al., 2013). The findings in another cellular experiment were aligned with the observations mentioned above, indicating that ginsenoside Rb1 effectively maintained the morphology and structure of neural cells in a hypoxic-induced co-culture model with microglia, diminished cell apoptosis, and suppressed the generation of NO and superoxide as well (Ke et al., 2014). The major function of astrocyte is to safeguard neurons from glutamate-induced excitotoxicity by scavenging excessive excitatory glutamate. Astrocytes captured glutamate via the glutamate transporter-1 (GLT-1) and further converted it into glutamine through the enzymatic action of glutamine synthetase (GS) (Nangaku et al., 2021). Zhang et al. suggested that ginsenoside Rb1 markedly decreased the number of reactive microglia and ameliorated neuroinflammation in LPS-treated mice (Zhang H. et al., 2021). Crucially, ginsenoside Rb1 modulated the activities of astrocyte and microglia via the GLT-1/GS system by elevating GLT-1 level and reversing the LPS-induced decrease in GS level, thus avoiding glutamate excitotoxicity (Zhang H. et al., 2021). Notably, these studies employed LPS-induced systemic inflammation mouse models and hypoxic co-culture systems to mimic inflammation microenvironment in the brain for evaluating the effects of ginsenosides on microglia. However, ischemic stroke animal models are still lacking.

#### 3.5.6 Paeoniflorin

Paeoniflorin, a water-soluble monoterpenoid glycoside separated from *Paeonia lactiflora* Pall., has extensive therapeutic effects, comprising anti-inflammation, anti-oxidation, anti-thrombosis, anti-convulsant, analgesic, neuroprotection, immunomodulation, and cognitive function enhancement (Zhou et al., 2020). Chen et al. found that paeoniflorin fostered the conversion of microglial phenotypes and reversed LPS-elicited inflammation, which were mediated by its regulatory actions on the NF-κB pathway (Chen et al., 2020). Tang et al. observed that paeoniflorin dampened the proliferation of microglia and produced a marked decrease in the generation of pro-inflammatory cytokines in rats with tMCAO. It also promoted neurogenesis and vasculogenesis after brain ischemic insult through suppressing JNK and NF-κB signaling pathways, thereby blocking inflammatory response and facilitating neurogenesis (Tang et al., 2021). In recent years, autophagy has been found to perform an essential role in normal cell function and homeostasis. Zhou et al. suggested that paeoniflorin attenuated neuroinflammation induced by microglia hyperactivation in LPS-treated BV2 microglia probably through reversing LPS-induced autophagy inhibition (Zhou et al., 2023).

### 3.6 Anthraquinones

#### 3.6.1 Emodin

Emodin is a natural derivative of anthraquinone, presenting in kinds of Chinese medicinal herbs, like *Rheum officinale*. Extensive evidence points out that emodin possesses multiple pharmacological properties, such as anti-cancer, anti-inflammation, anti-oxidation and anti-microbial activities (Dong et al., 2016). Jiang et al. found that emodin alleviated LPS/adenosine triphosphate (ATP)-stimulated pyroptosis in BV2 microglial cells (Jiang et al., 2023). Since pyroptosis is triggered by the activation of NLRP3 inflammasome and the pyroptosis-executing protein GSDMD pathway (Li S. et al., 2021), the findings further underscored that the suppressive effects of emodin on neuronal pyroptosis stemmed from its abilities to prevent the activity of the NLRP3 inflammasome and the cleavage of GSDMD. When HT-22 neurons co-cultured with BV2 microglia, emodin was found to protect HT-22 neurons from BV2 microglia pyroptosis-mediated toxicity (Jiang et al., 2023). Similarly, the study conducted by Li et al. suggested that emodin inhibited microglial pyroptosis and prompted M1 to M2 subtype transformation through suppressing the activation of microglial NLRP3 inflammasome (Li X. et al., 2024). Besides, emodin blocked the generation of NO and PGE2, as well as iNOS and COX-2 induced by LPS in the primary microglial cells, which was mediated by the enhancement of HO-1 and NADPH quinone oxidoreductase 1 (NQO1) via regulating the AMPK/Nrf2 signaling pathway (Park et al., 2016).

#### 3.6.2 Chrysophanol

Chrysophanol, the most common free anthraquinone species, is another important component separated from plants of the *Rheum genus*, exhibiting salutary effects in treating nervous system diseases (Su et al., 2020). Chrysophanol was found to hinder the generation of pro-inflammation mediators and cytokines in microglia through suppressing the activity of NF-κB and blocking the accumulative ROS. It also alleviated LPS-elicited mitochondrial fission via reducing dephosphorylation of dynamin-related protein 1 (DRP1) at the S637 site (Chae et al., 2017). Using a dMCAO mouse model and OGD or LPS-treated *in vitro* system, Liu et al. suggested that chrysophanol could regulate the polarization of microglia and blunt the expressions of inflammatory cytokines, thereby, enhancing the complexity of neurons and the density of neuronal spines. Further, the IL-6/STAT3 pathway was evidenced as a therapeutic target for anti-inflammation actions of chrysophanol (Liu X. et al., 2022).

### 3.7 Others

#### 3.7.1 Arctigenin

Arctigenin, a lignan compound extracted from Chinese medicinal herb *Arctium lappa* L., is extensively applied in inflammatory diseases (Li et al., 2023). Yuan et al. demonstrated that arctigenin blunted the activity of glial cells and downregulated the levels of pro-inflammatory mediators in LPS-induced systemic inflammation mice. Importantly, arctigenin treatment inhibited the triggering of the inflammation-associated TLR-4/NF-κB pathway. Furthermore, in BV2 microglial cells, arctigenin was observed to reverse the enhanced interaction between AdipoR1 and TLR4 and reduced the stability of the TLR4/CD14 complex, which in turn led to the suppression of TLR4-mediated signal transduction, thereby attenuating its downstream inflammatory response (Yuan et al., 2022).

#### 3.7.2 Ligustilide

Ligustilide is a characteristic phthalide component of *Angelica sinensis* and *Ligusticum chuanxiongs* with multiple neuroprotective activities (Wu et al., 2022). Kuang et al. reported that ligustilide could suppress the activation of astrocyte and microglia/macrophages, limit the invasion of neutrophils and T-lymphocytes from periphery to brain parenchyma and reduce the production of inflammatory mediators in rats with MCAO. Its neuroprotective actions were attributed to the inhibitory effects on the TLR4/peroxiredoxin 6 (Prx6) signaling pathway (Kuang et al., 2014).

### 3.8 Herb extracts

#### 3.8.1 Panax notoginseng saponins

Panax notoginseng saponins (PNS) are the major active compounds derived from herbal medicine *Panax notoginseng*, containing Ginsenoside Rb1, Ginsenoside Rg1, Notoginsenoside R1, Ginsenoside Rd and Ginsenoside Re (Huang et al., 2015), which have been extensively applied in treating cardiovascular and cerebrovascular disorders, especially stroke (Huang et al., 2015). Gao et al. reported that PNS suppressed the activation of microglia for ameliorating inflammatory response during the acute phase after stroke induced by photothrombosis. Moreover, PNS lowered the level of PKM2 in the nucleus of the activated microglia, concomitantly, blunted the hypoxia-inducible factor-1α (HIF-1α)/pyruvate kinase M2 (PKM2)/STAT3 pathway, which may underlie PNS’s inflammation-inhibitory effect in stroke (Gao et al., 2022). Network pharmacology screening revealed that MAPK signaling pathway was the key target pathway involved in the inhibitory effects of PNS on microglia-mediated inflammation. Molecular docking studies identified the binding sites of PNS to the MAPK pathway, revealing that PNS inhibited p39 and JNK activity and enhanced ERK1/2 phosphorylation through these interactions. These predictions were further validated in stroke animal models, providing experimental evidence to support the therapeutic potential of PNS for ischemic stroke (Duan et al., 2024).

#### 3.8.2 Salvianolic acids for injection

Salvianolic acids for injection (SAFI) is primarily composed of water-soluble constituents of the roots of *Salvia miltiorrhiza* Bunge, comprising salvianolic acids (B, D, Y), rosmarinic acid and alkannic acid (Li W. et al., 2018). It is a lyophilized powder for intravenous injection which has been authorized by the Chinese FDA in treating ischemic stroke (Lyu et al., 2019). Numerous studies suggested that neuroprotective properties of SAFI against ischemic injury are probably attributed to its anti-inflammation ability (Zhang et al., 2017). Ma et al. proposed that the neuroprotective effects of SAFI were mediated by facilitating the polarization of microglia from M1 to M2 subtype and by blocking NLRP3 inflammasome/pyroptosis axis, as demonstrated in both MCAO rats and OGD/R cell systems (Ma et al., 2021). Similarly, Zhuang et al. pointed out SAFI treatment dampened activated microglia-induced neuroinflammation, partly by blunting the TLR4/NF-κB signaling pathway (Zhuang et al., 2017).

**FIGURE 4:**
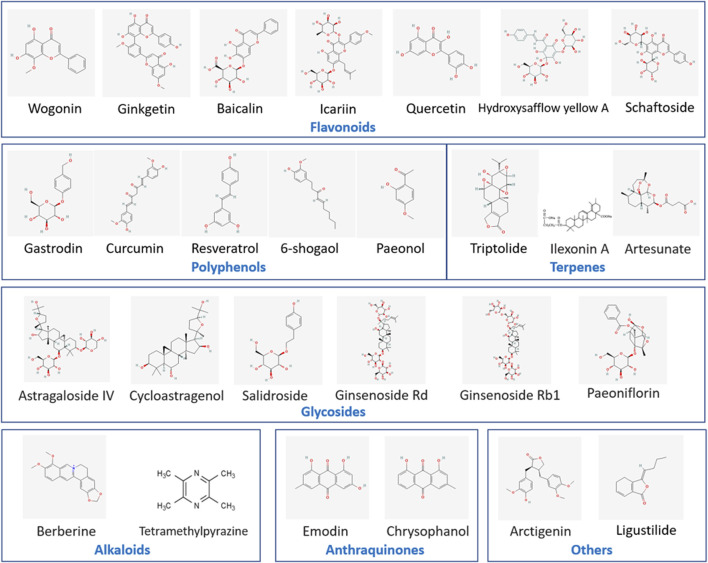
Chemical structural formulas of natural compounds in different categories. This figure lists the chemical structural formulas of natural compounds with the function of regulating microglial cell responses.

**FIGURE 5:**
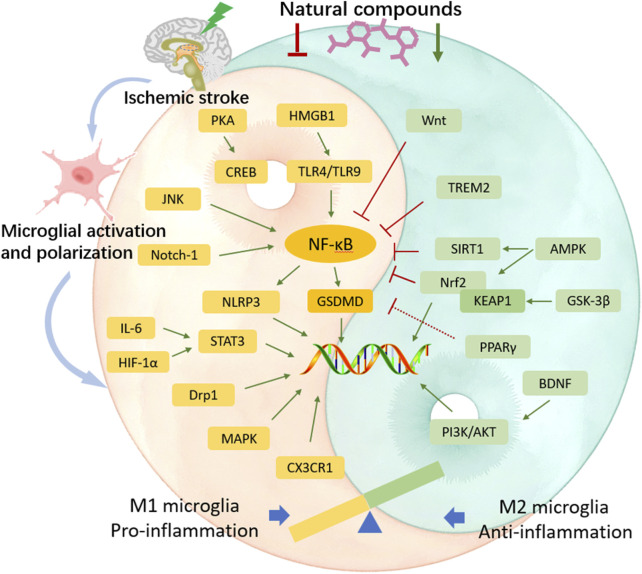
The regulatory effects of natural compounds from Chinere herbs on microglial activation after cerebral ischemia. After cerebral ischemic injury, microglia undergo rapid activation and polarization into two distinct phenotypes. M1-polarized microglia exacerbate the inflammatory response, causing further damage to brain tissue; whereas M2-polarized microglia exert anti-inflammatory effects and promote neuronal repair. Natural compounds can inhibit M1 phenotype-related pro-inflammatory signaling pathways, trigger M2 phenotype-related anti-inflammatory signaling pathways, to suppress the inflammatory response and ameliorate cerebral ischemic injury.

By collecting and analyzing relevant literature, we have identified the primary compounds isolated from traditional Chinese herbs that regulate microglia for stroke treatment as flavonoids, polyphenols, terpenes, alkaloids, glycosides, anthraquinones, and other herb extracts. Their chemical structural formulas are shown in Figure 4. Flavonoids, specific secondary metabolites from plants, are featured by two phenyl rings and a heterocyclic ring (Lu et al., 2024). The compounds that were evidenced to perform a beneficial role in microglial polarization include wogonin, ginkgetin, baicalin, icariin, quercetin, hydroxysafflow yellow A, and schaftoside (Table 2). Polyphenols, as natural antioxidants, possess a complex chemical structure with multiple hydroxyl groups on aromatic rings (Mamun et al., 2024). The presence of carboxyl and carbonyl groups determines their antioxidant activity (Li X. H. et al., 2022). In this article, the major polyphenolic compounds that exert anti-inflammatory effects through regulating microglia comprise gastrodin, curcumin, resveratrol, 6-shogaol, and paeonol (Table 3). Terpenes are the most abundant group of secondary metabolites in plants, including triptolide, Ilexonin A, artesunate, and others (Table 4). Their basic structure is made up of isoprene units. Based on the number of isoprene units, terpenes can be divided into monoterpenes, sesquiterpenes, diterpenes, triterpenes, and tetraterpenes (Araruna et al., 2020). Alkaloids are a common class of nitrogen-containing organic compounds, such as berberine and tetramethylpyrazine, which exist in various Chinese herbal medicines (Table 5). The compounds are characterized by complex ring structures with nitrogen elements, which serve as the key active group for alkaloids in treating ischemic stroke (Fan et al., 2024). Glycosides are sugar-containing compounds formed by an aglycone linked to one or more sugar moieties. Saponins are a common class of glycosides. The monosaccharide composition and the arrangement of sugar chains within the structure of glycosides can influence their diverse bioactivities (Thuan et al., 2024). Many glycosides have been validated to regulate microglia-associated neuroinflammation, including astragaloside IV, cycloastragenol, salidroside, ginsenoside Rd, ginsenoside Rb1, and paeoniflorin, and others. (Table 6). Anthraquinones, such as emodin and chrysophanol, are polycyclic compounds characterized by a 9,10-anthraquinone structure with three rings of A, B, and C. The side groups can be converted into the substitution patterns of hydroxyl groups, which provides anthraquinones with diverse biological activities (Wang P. et al., 2024) (Table 7). Arctigenin is a dibenzyl butyrolactone lignan from the medicinal plant *A. lappa*, and ligustilide is a natural phthalide existing in Angelica sinensis. Beyond that, herb extracts commonly used in the clinic, such as panax notoginseng saponins and salvianolic acids for injection, have remarkable anti-inflammatory effects (Table 8). Aforementioned compounds can regulate microglial response to ameliorate ischemia-induced inflammatory response through various signaling molecules and transduction pathways. These pathways are primarily associated with neuroinflammation, oxidative stress, endoplasmic reticulum stress, mitophagy, mitochondrial fission, neurotoxicity, the RAS system, embryo development, neurogenesis, angiogenesis, gut microbiota, and pyroptosis, which are critical processes in the pathogenesis of ischemic stroke (Figure 5). Moreover, changing the drug form of the compounds, using biomaterial-integrated drug delivery systems, and combining with some other small molecular drugs might exhibit superior efficiency in alleviating ischemic insults.

**TABLE 2:** Neuroprotective effects of flavonoids on microglial responses after ischemic stroke.

Natural compound	Experimental models		The main regulatory effects on microglial response	Mechanisms	Ref.
	*In vitro*	*In vivo*			
Wogonin	LPS/INFγ-induced BV2 microglial cells		PGE2, NO, iNOS, COX-2↓	ERK1/2 pathway↓, MEK1/2↓, Srs activation↓	Yeh et al. (2014)
Ginkgetin	OGD/R-stimulated primary microglial cells	MCAO rats	M1 microglia (Iba1+, CD16^+^)↓, M2 microglia (Iba1+, CD206+)↑, TNF-α, IL-1β↓, IL-4, IL-10↑	PPARγ signaling↑	Tang et al. (2022b)
Baicalin		LPS-induced neuroinflammatory mice	Microglia and astrocyte activation (Iba-1^+^, GFAP^+^)↓, IL-1β, TNF-α↓	NF-κB↓	Shah et al. (2020)
	LPS-induced BV2 microglial cells		NO, iNOS, IL-1β, COX-2 and PGE2↓	TLR4/MyD88/NF-κB and MAPK pathways↓, miR-155↓	Li et al. (2022a)
		BCCAO rats	Microglia activation (Iba-1^+^) ↓, iNOS↓, Arg-1↑, IL-1β, TNF-α↓	Wnt/β-catenin↑, NF-κB signaling↓	Xiao et al. (2023)
	LPS-stimulated BV2 microglial cells	LPS-stimulated neuroinflammatory mice	Microglia and astrocyte activation (Iba-1^+^, GFAP^+^)↓, IL-1β, TNF-α↓	SIRT1↑, HMGB1↓	Li et al. (2020b)
	OGD/R-stimulated BV2 microglial cells	MCAO mice	ROS↓; TNF-α, iNOS, MMP9, IL-1β, CD16, CD86↓; Arg-1, CD206↑	TREM2↑	Wang et al. (2024a)
Icariin	LPS-induced BV2 microglial cells		Microglia activation (Iba-1^+^) ↓, NO, IL-1β and IL-18↓	Nrf2↑, HO-1 and NQO1↑	Zheng et al. (2019)
	OGD/R-stimulated primary microglial cells		IL-1β, IL-6 and TNF-α↓	IRE1α/XBP1 pathway↓	Mo et al. (2021)
Quercetin	LPS/ATP-induced BV2 and primary microglial cells, dopaminergic neurons and hippocampal neurons cocultured with LPS and ATP stimulation		IL-1β, IL-6↓, microglial proliferation and phagocytosis↓, microglia activation (Iba1^+^, CD68^+^) ↓, ROS↓, pyroptosis↓, mitophagy↑	NF-κB↓, NLRP3 inflammasome↓, mitochondrial ROS stress↓	Han et al. (2021)
HSYA	OGD/R-stimulated primary microglial cells	MCAO rats	inflammation↓	TRL9↑, NF-κB pathway↓, IRF3↓	Gong et al. (2018)
	Microglial cells and primary neurons cocultured with LPS stimulation		microglia activation (CD11b^+^)↓,morphological changes↓, IL-1β, TNF-α, NO↓, BDNF↑	TLR4 pathway↓, NF-κB/MAPK/cytokine signaling↓	Lv et al. (2016)
Schaftoside	OGD-stimulated BV2 microglial cells		IL-1β, TNF-α, and IL-6↓	TLR4/Myd88 pathway↓, Drp1↓, mitochondrial fission↓	Zhou et al. (2019)

**TABLE 3:** Neuroprotective effects of polyphenols on microglial responses after ischemic stroke.

Natural compound	Experimental models		The main regulatory effects on microglial response	Mechanisms	Ref.
	*In vitro*	*In vivo*			
Gastrodin	LPS-stimulated BV2 or primary microglia	Three-day postnatal rats treated with LPS	iNOS, TNF-α↓, cyclin-D1, Ki67↓, proliferation in BV2 microglia and brain microglia↓	GSK-3β↓, Wnt/β-catenin pathway↓	Yao et al. (2019)
	OGD-stimulated BV2 microglial cells		IL-1β, TNF-α↓, BDNF↑	MAPK↓	Lv et al. (2021)
	LPS-induced BV2 microglial cells	Postnatal rats with hypoxic-ischemia brain damage	NOX-2, iNOS and TNF-α↓	ACE, AT1↓, caspase-3↓, AT2 and SIRT3 pathway↑	Liu et al. (2018a)
	LPS-induced BV2 microglial cells	LPS-induced inflammation in postnatal rats	IL-1β, IL-6, IL-23, TNF-α and NO↓	Notch-1 pathway↓, MAPK↓	Yao et al. (2022)
Curcumin	LPS/IFN-γ-stimulated BV2 microglial cells	dMCAO mice	M1 microglia (Iba1+, CD16^+^)↓, M2 microglia (Iba1+, CD206+)↑, TNF-α, IL-6, IL-12p70↓		Liu et al. (2017b)
	primary microglial cells treated with LPS and ATP	MCAO mice	GSDMD+, caspase-1+ in Iba1+ microglia/macorphage↓, cleaved caspase-1, NLRP3, IL-1β, IL-18↓	NF-κB/NLRP3 pathway↓	Ran et al. (2021)
NPcurcumin		tMCAO mice	M1 phenotype (Iba1+, CD68^+^)↓, IL-1β, TNF-α↓, apoptosis↓, tight junction proteins↓		Wang et al. (2019b)
Curcumin gel	OGD-stimulated BV2 microglial cells	Photothrombic stroke model in mice	ROS↓; CD16, IL-1β↓; CD206, TGF-β↑; Iba-1/iNOS↓; Iba-1/CD206↑; PSD-95↑	ROS-NFκB pathway↓; p47-phox and p67-phox translocation↓	()
Resveratrol		Rats with 4-VO	Microglial activation (CD11b/c+)↓, astrocyte activation (GFAP+) ↓	GLT-1↑	Girbovan and Plamondon (2015)
		tMCAO rats	Micorglial activation (Iba1+)↓, M1 microglia (iNOS+)↓, M2 microglia (Ym1/2+, CD206+)↑	Nrf2↑, regulating miR-450b-5p/KEAP1 axis	Liu et al. (2023b)
MS275+resveratrol	Primary mixed glial cells exposed to NCM-OGD	pMCAO mice	Nos2, IL-1β, IL-6↓, Mrc1, Ym1, iNOS↓, CD68↓ 1 day after pMCAO, Ym1, Arg1, CD32↑ 7 days after pMCAO, microglia activation (Iba1+)↓, LDH↓	The binding of RelA to Nos2 promoter↓	Mota et al. (2020)
6-shogaol	LPS-induced BV2 microglial cells	MCAO mice	NO, iNOS↓, TNF-α, IL-6↓, microglial activation (Iba1+)↓		Gaire et al. (2015)
	LPS-induced BV2 microglial cells		TNF-α, IL-1β, IL-6 and PGE2↓	NF-κB↓,PPAR-γ↑	Han et al. (2017)
Paenonal	BV2 microglial cells treated with LPS/IFN-γ	LPS-injected mice	NO, iNOS, COX-2, ROS↓, cell migratory activity↓, microglial activation (Iba1+)↓	AMPK/GSK3↑	Lin et al. (2015)
	LPS-induced N9 microglial cells		NO, iNOS↓, IL-1β, PGE2↓, COX-2↓	NF-κB↓, MAPK pathway↓, TLR4 pathway↓	He et al. (2016)

**TABLE 4:** Neuroprotective effects of terpenes on microglial responses after ischemic stroke.

Natural compound	Experimental models		The main regulatory effects on microglial response	Mechanisms	Ref.
	*In vitro*	*In vivo*			
Triptolide	LPS-stimulated primary rat microglial cells and BV2 cells, MN2D and SH-SY5Y cells treated with conditioned medium from LPS-induced microglia		NO and iNOS synthesis↓	EP2/PKA pathway↓	Zhang et al. (2015)
	LPS-stimulated BV-2 microglial cells	MCAO/R mice	IBA-1^+^ and iNOS^+^ cells↓; Arg-1^+^ cells↑; TNF-α, IL-1β↓, HT-22 cell viability↑; HT-22 cell apoptosis↑	CTSS/Franctalkine/CX3CR1 signaling pathway↓	Zhou et al. (2024)
Triptolide + Ki20227		C57BL/6 mice with focal ischemic stroke induced by photochemical induction techniques	Synaptic protein expressions↑; dendritic spins density↑; microglia activation (Iba1^+^) ↓	CSF1R signal↓; autophagy↑; BDNF-Akt pathway↑	Du et al. (2020)
Ilexonin A		MCAO rats	Microglia activation (Iba-1^+^)↓ at each time point; astrocyte activation (GFAP^+^)↑ at 1, 7 days, ↓at 14 days; VEGF, Flk-1, Nestin↑		Xu et al. (2016)
		MCAO rats	Microglia activation (Iba-1^+^) and astrocyte activation (GFAP^+^)↑ in the hippocampus; nestin↑; TNF-α, IL-1β↓		Xu et al. (2020a)
Artesunate	BV2 microglia treated with LPS + IFNγ		PGE2↓; mPGES-1, COX-2↓; TNF-α, IL-6 ↓	NK-κB and p38 MAPK signaling↓	Okorji and Olajide (2014)
		dMCAO mice	MPO↓; microglia activation (Iba-1^+^)↓; TNF-α, IL-1β↓	NF-κB pathway↓	Liu et al. (2021b)

**TABLE 5:** Neuroprotective effects of alkloids on microglial responses after ischemic stroke.

Natural compound	Experimental models		The main regulatory effects on microglial response	Mechanisms	Ref.
	*In vitro*	*In vivo*			
Berberine		tMCAO rats	CD86-positive microglia↓; CD163-positive cells↑; IL-1, IL-6, TNF-α↓; IL-4, IL-10↑; vagal afferent nerve activity↑; intestinal H_2_S production↑	TRPV1 receptors-dependent	Ni et al. (2022)
	LPS-induced BV2 microglial cells	tMCAO mice	M1 phenotype markers (IL-1β, CD32, TNF-α) ↓; M2 phenotype markers (CD206, Arg-1, Ym1/2) ↑; CD16^+^/Iba1^+^ microglia↓; CD206^+^/Iba1^+^ microglia↑; angiogenesis↑	AMPK↑	Zhu et al. (2019b)
		Gerbils with global ischemia	CD11b, GFAP↓; capase-3↓; apoptosis↓; cytochrome *c*↓	PI3K/Akt↑; Bax/Bcl-2↓	Kim et al. (2014)
Tetramithylpyrazine		Rats with permanent cerebral ischemia	Leukocyte intracerebral infiltration↓; activated macrophages/microglia (CD45^+^/CD11b^+^) ↓	JNK/AP-1 pathway↓; Nrf2, HO-1↑	Kao et al. (2013)
	LPS-induced N9 microglial cells		NO, iNOS↓; ROS↓	NF-κB↓; MAPK↓; Akt↓	Liu et al. (2010)
	LPS + IFN-γ-stimulated BV2 microglia	MCAO rats	NG2^+^, Ki67^+^/NG2^+^, CNPase^+^, Ki67^+^/CNPase^+^ cells↑; Iba1^+^ and Iba1^+^/CD16^+^ cells↑; IL-6↓; IL-10↑	JAK2/STAT3 pathway↑; STAT1↓; GSK3/NFκB pathway↓	Feng et al. (2023)

**TABLE 6:** Neuroprotective effects of glycosides on microglial responses after ischemic stroke.

Natural compound	Experimental models		The main regulatory effects on microglial response	Mechanisms	Ref.
	*In vitro*	*In vivo*			
Astragaloside IV		tMCAO rats	microglia activation (Iba1^+^) ↓; M1 microglia (CD86^+^, CD16/32^+^, iNOS^+^)↓; TNF-α, IL-1β, IL-6↓; M2 microglia (Arg-1^+^, YM1/2^+^, CD206^+^)↑; IL-10, TGF-β↑; BrdU^+^/NeuN^+^ and BrdU^+^/GFAP^+^ cells↑; BrdU^+^/vWF^+^ cells↑; CD206^+^/BDNF^+^ and CD206^+^/IGF1^+^ cells↑; VEGF, IGF-1, BDNF↑	PPAR-γ pathway↑	Li et al. (2021a)
	OGD/R-induced primary rat microglia cells	MCAO rats	The survival rate of primary rat microglia cells↑; Iba-1↓	NLRP3 inflammasome pathway↓; LOC102555978↓; miR-3584-5p↑	Gao et al. (2024)
		BCCAO mice	microglia activation (Iba1^+^) ↓; TNF-α, IL-1β↓; MDA, ROS↓; SOD↑	TLR4/NF-κB pathway↓; NLRP3 inflammasome↓; cleaved caspase-1↓	Li et al. (2017)
	LPS-stimulated BV2 and primary microglial cells		NO, IL-6, TNF-α↓	ERK↑; NRF2/HO-1 pathway↑	Li et al. (2018a)
	LPS + IFN-γ-stimulated BV2 microglial cells	MCAO rats	C16^+^/IBA1^+^ cells↓; Arg1^+^/IBA1^+^↑; CD16, CD86, iNOS, IL-1β, IL-6↓; CD206, BDNF, TGF-β1, IL-10↑; glycolytic key proteins↓	AMPK↑; mTOR/HIF-1α signaling pathway↓	Li et al. (2024a)
Cycloastragenol	LPS-stimulated BV-2 mouse microglial cells	MCAO mice	TNF-α, IL-1β, IL-6↓; iNOS, NO, COX-2↓; ROS↓; CD206↑; microglia activation (Iba1^+^) ↓; M1 microglia (CD16/32^+^)↓; M2 microglia (CD206^+^)↑	NF-κB↓; Nrf2/HO-1 pathway↑	Chen et al. (2022b)
		MCAO mice	MMP9↓; ZO-1, occluding↑; BBB disruption↓; TNF-α, IL-1β↓; microglia activation (Iba1^+^) ↓	SIRT1↑; p53 acetylation↓; Bax/Bcl-2 ratio↓; NF-κB↓	Li et al. (2020a)
Salidroside	BV-2 microglia cultured in hypoxia	Mice exposed to hypobaric hypoxia	Occluding, claudin-5↑; Iba-1↓; GSH↑; SOD, MDA↓; IL-18, IL-6, TNF-α↓	GSK3β↑, Nrf-2↑	Zhang et al. (2024b)
	Deferoxamine-stimulated BV2 microglial cells		LDH, ROS, HIF-1α, NF-κB p65, TNF-α, IL-1β, IL-6↓; inverting cell energy metabolism		Fan et al. (2022)
	Neuron-microglia cocultures exposed to OGD, microglia-oligodendrocyte cocultures, primary cortical neurons subjected to OGD	Mice with MCAO	Microglia activation (Iba-1^+^)↓; M1 microglia/macrophages (Iba-1^+^, CD16/32^+^)↓; microglia/macrophages (Iba-1^+^, CD206^+^)↑; LDH↓; CD16 and iNOS↓; CD206 and Arg1↑; IL-1β, IL-2, IL-6, IL-8, TNFα↓; oligodendrocyte differentiation↑		Liu et al. (2018b)
	OGD/R-induced BV2 cells	MCAO rats	LDH↓; apoptosis↓; TNF-α, IL-6, IL-8↓	TLR4/NF-κB pathway↓; NLRP3 inflammasome↓	Liu et al. (2021a)
		MCAO rats	CD11b↓; CD14, CD44, TNF-α, IL-6, IL-1β, iNOS↓	PI3K/Akt pathway↑; HIF↑	Wei et al. (2017)
Ginsenoside Rd	OGD-, LPS-induced BV2, primary microglial cells		Microglia activation (Iba-1^+^)↓; IL-1β, IL-6, IL-18, TNF-α, IFN-γ↓	NF-κB activation↓; proteasome activities↓	Zhang et al. (2016b)
Ginsenoside Rb1		Mice with systemic LPS-induced inflammation	Iba1↓; morphological activation of microglia↓; TNF-α, IL-6, IL-1β↓; COX-2↓		Lee et al. (2013)
	Cortical neuron-N9 microglia hypoxic coculture system		Neuronal apoptosis↓; caspase-3↓; TNF-α, NO, superoxide↓		Ke et al. (2014)
		Mice with LPS-induced inflammation	Microglia activation (Iba-1^+^)↓; IL-1β↓	GLT-1, GS↑	Zhang et al. (2021a)
Paeoniflorin	OGD/R-induced BV-2 microglial cells	tMCAO rats	Microglial cell proliferation↓; Iba1↓; IL-1β, TNF-α, IL-6↓; microglial viability↓; neurogenesis and vasculogenesis↑	JNK/NF-κB signaling↓	Tang et al. (2021)
	LPS-induced BV-2 microglial cells		TNF-α, IL-1β, IL-6, IFNγ↓; IL-4, IL-10↑, SOD, GSH↑; ROS, MDA↓; M1 microglia (iNOS^+^, CD32^+^)↓; M2 microglia (Ym1^+^)↑	NF-κB pathway↓	Chen et al. (2020)
	LPS-induced BV-2 microglial cells		LC3-II↑; p62↓; IL-1β, TNF-α↓		Zhou et al. (2023)

**TABLE 7:** Neuroprotective effects of anthraquinones on microglial responses after ischemic stroke.

Natural compound	Experimental models		The main regulatory effects on microglial response	Mechanisms	Ref.
	*In vitro*	*In vivo*			
Emodin	LPS/ATP-induced BV2 microglial cells, BV2 and HT-22 cocultures stimulated by LPS/ATP		Pyroptosis↓; TNF-α, IL-1β, IL-18↓	NLRP3 inflammasome, GSDMD↓	Jiang et al. (2023)
	OGD/R-induced BV-2 microglial cells	tMCAO rats	M1 microglia (CD32^+^/Iba1^+^)↓; M2 microglia (CD206^+^/Iba1^+^)↑; CD16, CD32, iNOS↓; CD206, Arg1, CCL-22↑; GSDMD↓	NLRP3 inflammasome↓	Li et al. (2024c)
	LPS-induced primary microglia		HO-1, NQO1↑; NO, PGE2↓; iNOS, COX-2↓; TNF-α, IL-6↓	AMPK/Nrf-2↑; NF-κB↓	Park et al. (2016)
Chrysophanol	LPS-induced BV2 microglial cells	dMCAO mice	Microglia activation (Iba-1^+^)↓; pro-inflammatory phenotype marker (CD16/32)↓; IL-6↓; the neuron complexity and the spine density	IL-6/JAK/STAT3 pathway↓	Liu et al. (2022b)
	LPS-induced BV-2 murine microglial cells		NO, IL-1β, IL-6, TNF-α↓; ROS↓; NF-κB↓; mitochondrial fission↓	Regulating MAPK and NF-κB pathway; Drp1 dephosphorylation↓	Chae et al. (2017)

**TABLE 8:** Neuroprotective effects of other compounds and herb extracts on microglial responses after ischemic stroke.

Natural compound	Experimental models		The main regulatory effects on microglial response	Mechanisms	Ref.
	*In vitro*	*In vivo*			
Other compounds					
Arctigenin	LPS-induced BV-2 microglial cells	Mice with LPS-induced inflammation	Synaptic density↑; Iba-1, GFAP↓; TNF-α, IL-1β, IL-6↓	AdipoR1↓; NF-κB↓; TLR4/CD14↓	Yuan et al. (2022)
Ligustilide		MCAO rats	TNF-α, IL-1β, ICAM-1, MMP-9, IFN-γ, IL-17↓; IL-10↑	Prx6↓; TLR4 signaling↓	Kuang et al. (2014)
Herb extracts					
Panax notoginseng saponins		Mice with photothrombotic stroke	Microglia activation (Iba-1^+^)↓; TNF-α, IL-1β↓	PKM2↓; HIF-1α/PKM2/STAT3 signaling↓	Gao et al. (2022)
		MCAO rats	iNOS, TNF-α, IL-1β↓	P38, JNK↓; ERK1/2 phosphorylation↑	Duan et al. (2024)
Salvianolic Acids for Injection	Primary neurons and primary microglia cocultures stimulated by OGD/R	MCAO/R rats	Iba-1^+^/CD16^+^ cells↓; Iba-1^+^/CD206^+^ cells↑; caspase-1, IL-1β↓	NLRP3 inflammasome/pyroptosis axis↓	Ma et al. (2021)
	LPS-stimulated BV-2 microglia	MCAO rats	Microglia activation (Iba-1^+^)↓; IL-1β, IL-6, TNF-α, NO↓	TLR4/NF-κB↓	Zhuang et al. (2017)

## 4 Clinical application of natural compounds that modulate microglial response

In recent years, numerous clinical trials have been conducted on ischemic stroke, focusing on the therapeutic effectiveness and safety of the compounds isolated from traditional Chinese herbs. These compounds have been proven to participate in the regulation of microglial response in preclinical studies. A meta-analysis included a total of 14 randomized control trials (RCTs), comprising 1309 individuals suffering from acute ischemic stroke, showed that the combination of injectable salvianolic acids with the conventional treatment demonstrated superior therapeutic outcomes compared to the conventional treatment alone (Lyu et al., 2019). This superiority was embodied in the enhanced total effective rate and the recovery in neurological impairments, as well as the improvement in activities of daily living. Drug side effects in all studies were minor and transient, and the symptoms were disappeared quickly upon discontinuation of the medication (Lyu et al., 2019). For assessing the therapeutic effectiveness and safety profile of ginsenoside Rd in clinical practices, Zhang et al. carried out a pooled analysis (Zhang G. et al., 2016). The data collection process comprised two phases. In the first phase, data were gathered from 199 cases with acute ischemic stroke, and in the second phase, data were from 390 cases. By applying modified Rankin Scale (mRS) score on day 90 following stroke, the findings revealed that ginsenoside Rd effectively alleviated the degrees of disability in patients, and by applying NIH Stroke Scale (NIHSS) and Barthel Index (BI) scores on days 15 and 90 following stroke, ginsenoside Rd exhibited an improvement in neurological deficits (Zhang G. et al., 2016). A total 47 adverse events were recorded in the ginsenoside Rd group, indicating its low incidence of adverse reactions in clinical practices (Zhang G. et al., 2016). Luo et al. conducted a meta-analysis including 17 clinical studies, involving 1670 individuals with acute ischemic stroke, to assess the therapeutic effects of berberine (Luo et al., 2023). The findings revealed that berberine held suppression abilities on neuroinflammation and could be applied as an adjuvant agent for ischemic stroke. And berberine in combination with the conventional treatment exhibited superior outcomes to the only conventional treatment (Luo et al., 2023). These positive outcomes were manifested in the improvements in inflammatory markers, indicators of immune function, related biomarkers and atherosclerosis of the carotid artery (Luo et al., 2023). Available clinical data suggested that modulating microglia-driven inflammatory responses using natural compounds might be an innovative option for ischemic stroke. Since microglia perform essential roles in modulating the innate immune response (Xu S. et al., 2020), it is necessary to note that inappropriate or excessive inhibition of their activation may potentially influence or destroy other defensive mechanisms in the immune system. Owing to the intricacy of the immune system and the fact that natural compounds possess multiple action targets, it is plausible that compounds may cause some unanticipated adverse reactions during the management of ischemic stroke. Herein, conducting large-scale, long-term, and well-designed clinical trials with rigorous follow-up is imperative for further investigating safety profiles of these compounds. Such trials must evaluate not only the therapeutic benefits but also the toxicities and side effects of the compounds to ensure that any potential benefits outweigh the risks.

## 5 Conclusion and perspectives

**FIGURE 6:**
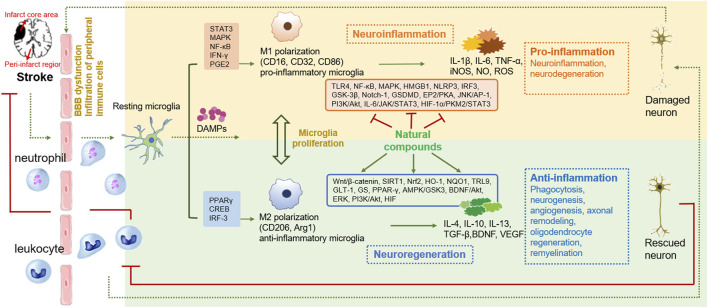
The effects of natural compounds on microglial activation and polarization in ischemic stroke. After the onset of brain ischemia, resting microglia become activated and change their morphology in the amoeboid shape. Activated microglia may be polarized into pro-inflammatory M1 phenotype or anti-inflammatory M2 phenotype. The polarization of M1 state is mediated by STAT3, MAPK, NF-κB, IFN-γ and PGE2 signaling, etc., and leads to the production of pro-inflammatory cytokines. The polarization of M2 state involves PPARγ, CREB and IRF-3 signaling, etc. Natural compounds could inhibit the activation, proliferation and M1 phenotypic polarization of microglia, while promote M2 phenotypic polarization to mitigate inflammatory response for protecting brain blood barrier, resulting in neuroprotection and neurorestoration.

Strategies that inhibit microglia-mediated detrimental inflammatory response while enhancing their inflammation-suppressive abilities serve as effective therapy methods against ischemic stroke. Regulating microglial polarization by natural compounds from traditional Chinese herbs is considered as a crucial aspect in attenuating ischemic injury (Figure 6). Although natural compounds hold great potential to ameliorate neuroinflammatory response after stroke, research on microglia and their regulation in ischemic stroke still has limitations. For instance, cell surface markers used to distinguish between M1 subtype and M2 subtype can also be expressed by other immune cells. This overlap complicates the specific identification and isolation of microglial phenotypes. Appropriate methods and optimized technologies for segregating two subtypes of microglia would facilitate further differentiation of their pro- or anti-inflammatory characteristics. In addition, multiple factors are involved in brain ischemia and reperfusion injury, including the severity of the ischemic episode, the timing of therapeutic intervention, the approach for reperfusion treatment, as well as the presence of complications, especially the duration of the ischemic period. It is imperative to closely mimic the pathological conditions associated with these factors. Notably, animal models and related *in vivo* studies are still insufficiently designed to simulate the ischemic stroke conditions specific to elderly patients. For better addressing the aforementioned issues, the utilization of single-cell analysis, omics-based technology, and cerebral imaging methods is needed to detect and identify more sensitive microglial markers and specific targets for drugs. Besides, various influencing factors and pathological states should be considered when studying effects of natural compounds on microglia. Moreover, developing optimized animal-based and cellular stroke models simulating the intricate cerebral microenvironment is urgently needed. Further, when using natural compounds to treat stroke, it is essential to remain vigilant about potential unexpected adverse reactions, as these could interact with other pathological processes or signaling networks, thus influencing therapeutic effects and the judgement of outcomes.

Though large number of preclinical studies have been conducted on the treatment of ischemic stroke with natural compounds, their clinical applications still encounter challenges. On one hand, unclear mechanisms and action targets of compounds in brain ischemia restrict new drugs development. Proper *in vivo* methods need to be established for finding drug targets directly in the real physiological environment (Zhang et al., 2025). On the other hand, complex chemical components contained in Chinese herbal extracts pose difficulties for the isolation of monomer components with pharmacological activity and quality control of drugs (Zhu et al., 2022). Diverse factors, like sources of herbs, cultivation processes, collection and processing will affect the content of the active components within the medicinal herbs. Besides, significant first-pass effect, drug stability, and inappropriate administration routes may reduce the bioavailability of natural plant drugs. Novel drug delivery methods, such as nasal drug delivery (Rajput et al., 2022), biosynthetic drug delivery systems, such as nanoparticle encapsulation (Wang Y. et al., 2019), change in drug dosage forms, and drug purification processes, will be enable drugs to target the brain more precisely and improve their bioavailability. Importantly, issues regarding the toxic and side effects of natural compounds remain to be addressed before clinical practices. For example, excessive use of glycyrrhizic acid (GA) and glycyrrhetinic acid (GRA) may cause corticosteroid-like adverse reactions (Makino, 2021). Saikosaponins, the main active components of bupleurum chenense, have been reported to induce hepatotoxicity, neurotoxicity, hemolysis, and cardiotoxicity (Zhou et al., 2021). Besides, the potential adverse reactions arising from the interactions between natural compounds and other drugs need to be taken seriously. Herein, future investigations of natural compounds require the verification of long-term effects and a deeper exploration on pharmacological effects, including bioavailability, safety and toxicity, biosynthesis, drug delivery systems, and potential synergistic effects when combined with other compounds or Western medicine. Classical Chinese medicine formulas are widely applied in clinical practice. In recent years, there have been numerous reports on the regulation of microglial polarization by classical formulas to relieve cerebral ischemia. For instance, Buyang Huanwu Decoction suppressed microglia M1 polarization while simultaneously promoting microglia M2 polarization via AMPK pathways-mediated energy transporters and NF-Κb/CREB pathways (Li et al., 2024b). Huangqi Guizhi Wuwu Decoction modulated M2 microglia polarization and synaptic plasticity via regulating SIRT1/NF-κB/NLRP3 pathway (Ou et al., 2023). However, the components of Chinese herbal decoctions are complex, containing a variety of chemical substances, making it difficult to accurately analyze all of them, which affects the determination of quality control standards and the identification of the action targets of their active components.

It is worth noting that emerging evidence shows that exosome from different sources can regulate microglia polarization, which is mediated by exosomal miRNA cargo. Given the ability of exosomes to cross the blood-brain barrier, the use of endogenous exosomes or exosomes as carriers to transport some drug molecules to promote M2 polarization during brain ischemia offers new opportunities for stroke treatment (Wan et al., 2022). Recent study reported that M2 microglia-derived exosomes promoted the communication between M2 microglia and oligodendrocyte precursor cells, suggesting a promising therapeutic strategy for white matter repair in stroke (Li Y. et al., 2022). However, the precise number of exosomes transferred into the brain, the distribution and metabolism of exosomes, and key techniques for enhancing exosome targeting efficiency, are important issues that require deeper investigation (Li Y. et al., 2022; Ikeda et al., 2024). Considering the intricate pathogenesis of ischemic stroke, as well as the multi-targeting and multi-effecting properties of natural compounds, further studies on microglial biological properties and the regulation of microglial polarization in cerebral ischemia are not only necessary to identify novel natural compounds with optimal neuroprotective effects, but also to provide evidence that supports new clinical drug development targeting microglia-mediated neuroinflammation for ischemic stroke.
