# Supplementary material for: Targeting microglia polarization with Chinese herb-derived natural compounds for neuroprotection in ischemic stroke
Source: Front Cell Dev Biol. 2025 Jun 10;13:1580479. doi: 10.3389/fcell.2025.1580479 (PMC12185465; doi:10.3389/fcell.2025.1580479)
Supplement: Supplementary file 1 [file Table1.docx]

**Supplementary Table S1 Representative traditional Chinese medicinal herbs containing natural compounds for regulating microglial response**

| **Botanical name** | **Chinese name** | **Functions** | **Application in traditional Chinese medicine** | **Active compounds regulating microglial response** | **Ref.** |
| --- | --- | --- | --- | --- | --- |
| *Angelica sinensis* (Oliv.) Diels | Dang Gui | Invigorating blood, tonifying blood, activating blood circulation, dispersing cold, alleviating pains | Ischemic stroke, anemia, menstrual disorders, dizziness, palpitations, abdominal pain | Ligustilide | (Giacomelli et al., 2017; Hong et al., 2021) |
| *Artemisia annua. L* | Qing Hao | Dispersing deficiency heat, alleviating jaundice, eliminating malaria, clearing summer heat, cooling blood | Malaria, cancer, diabetes, pulmonary tuberculosis | Artesunate | (Wang et al., 2023) |
| *Arctium lappa* L. | Niu Bang Zi | Dispelling wind-heat, clearing lungs to promote sweating, detoxifying, alleviating throat pain | Cancer, diabetes, cold, fever, cough | Arctigenin | (Jin et al., 2023) |
| *Astragalus mongholicus* Bunge | Huang Qi | Nourishing qi and strengthening the exterior, healing sores and promoting muscle, improving water metabolism, immunostimulant, nourishing blood, diuretic, tonic, expectorant, detoxicating | Weakness, anemia, fever, vertigo, palpitations, fatigue syndrome, decreased appetite, neurodegenerative diseases, viral and bacterial infections, cancer | Astragaloside IV, Cycloastragenol | (Dong et al., 2022; Yu et al., 2022d) |
| *Carthamus tinctorius* L. | Hong Hua | Invigorating blood circulation | Ischemic stroke, coronary artery disease, angina pectoris, hypertension, gynecologic disorders | Quercetin, Kaempferol, Hydroxysafflow yellow A | (Zhang et al., 2016c; Yu et al., 2022b) |
| *Ligusticum chuanxiong* Hort. | Chuan Xiong | Invigorating qi and blood, circulation for eliminating stasis | Ischemic diseases, menstrual disorders, vertigo, dizziness, headache, anemia | Tetramethylpyrazine | (Lin et al., 2022) |
| *Coptis chinensis* Franch. | Huang Lian | Cooling and detoxification, dispelling dampness, anti-inflammation | Bacillary dysentery, high fever coma, toothache, diabetes, pertussis, sore throat, aphtha, eczema, diarrhea | Berberine | (Wang et al., 2019a) |
| *Curcuma Longa* Linn. | Jiang Huang | Promoting blood circulation, removing blood stasis, modulating qi downward, stopping bleeding | Vascular disorders, hypertension, diabetes, cardiovascular diseases, inflammatory bowel disease, cancer, acute kidney injury | Curcumin | (Cai et al., 2022; Weng and Goel, 2022) |
| *Epimedium brevicornu* Maxim. | Yin Yang Huo | Tonifying kidneys and warming yang | Vascular disorders, hypertension, osteoporosis, osteoarthritis | Icariin | (Wang et al., 2018; Zhang et al., 2022) |
| *Gastrodia elata* Blume. | Tian Ma | Extinguishing wind, relieving convulsions | Vertigo, dizziness, headache, stroke, numbness, neurodegenerative diseases | Gastrodin | (Zhu et al., 2019a; Sun et al., 2023) |
| *Ginkgo biloba* L. | Yin Xing Ye | Promoting blood circulation, reducing phlegm, clearing poison, anti-inflammation, dredging collaterals and relieving pain, stabilizing the lungs and relieving dyspnea | Angina pectoris and coronary heart disease, cerebrovascular disease, Alzheimer’s disease, atherosclerosis, cancer, asthma, non-alcoholic fatty, inflammatory and intestinal disease | Ginkgetin | (Liu et al., 2022c) |
| *Herba Desmodii Styracifolii* | Guang Jin Qian Cao | Clearing heat and promoting diuresis, dispersing dampness and alleviating jaundice, stopping cough and expelling phlegm, relieving swelling and removing toxins, stone elimination | Urolithiasis, jaundice hepatitis, liver cirrhosis, cholelithiasis | Schaftoside | (Liu et al., 2017a; Liu et al., 2020c) |
| *Ilex pubescens Hook. et Arn.* | Mao Dong Qing | Reducing internal heat, cooling blood and stopping bleeding, improving circulation, reducing swelling and removing pain | Inflammatory diseases, coronary artery disease, angina, vasculitis, brain ischemic injury | Ilexonin A | (Zhang et al., 2016a; Jiang et al., 2019) |
| *Polygonum cuspidatum Sieb. et Zucc.* | Hu Zhang | Eliminate heat and dampness, remove phlegm, disperse blood stasis | Inflammatory conditions, diabetes, gout, cough, lung diseases, external injuries, hypolipidemia, cancer, acute kidney injury | Resveratrol | (Yang et al., 2024) |
| *Paeonia lactiflora* Pall. | Shao Yao | Tonifying blood, regulating menstruation, harmonizing the excessive liver yang and preserving yin, relieving pain | Autoimmune diseases, depressive symptoms, tumor, inflammation | Paeonol, Paeoniflorin | (Wang et al., 2021) |
| *Panax ginseng* C.A.Meyer | Ren Shen | Nourishing qi and blood, calming | Insomnia, nervous disorders, cardiovascular disease, cancer, immune modulation, regulation of sugar and lipid metabolism, impotence | Ginsenoside Rb1, Ginsenoside Rd | (Chen et al., 2022c) |
| *Panax notoginseng* (Burkill) F.H.Chen | San Qi | Activating blood circulation, stopping bleed | Coronary diseases, ischemic stroke, atherosclerosis, hemoptysis and hemostasis, bleeding, blood stasis | Ginsenoside Rb1, Ginsenoside Rd, Panax notoginseng saponins | (Liu et al., 2022a) |
| *Rheum palmatum L.* | Da Huang | Cooling heat and relieving internal heat, eliminating constipation, clearing heat and removing toxins, removing blood stasis | Inflammatory diseases, constipation, cancer, depression, bacterial infection, chronic kidney disease | Chrysophanol, Emodin | (Gu et al., 2022) |
| *Rhodiola rosea* Linn. | Hong Jing Tian | Nourishing blood, invigorating qi, promoting blood circulation | Altitude disease, hypoxia, high altitude hypoxia, ischemic stroke, cardiovascular diseases, depression, fatigue | Salidroside | (Fan et al., 2020; Pu et al., 2020) |
| *Salvia miltiorrhiza* Bunge | Dan Shen | Removing blood stasis, activating blood circulation, regulating menstruation, calming | Cardiovascular diseases, neurasthenic, insomnia,  gynecologic disease | Tanshinone IIA, Salvianolic acids | (Wang et al., 2017) |
| *Scutellaria baicalensis* Georgi | Huang Qin | Cooling, nourishing yin, dispersing dampness | Lung diseases, diarrhea, dysentery, hypertension, bleeding, respiratory infections | Wogonin, Baicalein, Baicalin, Chrysin | (Liao et al., 2021) |
| *Tripterygium wilfordii* Hook. F. | Lei Gong Teng | Dispelling wind, eliminating dampness, activating blood circulation, facilitating detumescence, relieving pain | Lupus disease, cancer, rheumatoid arthritis, nephrotic syndrome, Behcet’s disease, neurodegeneration, atherosclerosis, fibrosis | Celastrol, Triptolide | (Chen et al., 2018) |
| *Zingiber officinale* Roscoe | Sheng Jiang | Alleviating exterior syndrome, dispelling cold, warming the Middle Jiao | Colds, nausea, emesis, headaches, neurodegeneration, cardiovascular disease, obesity, diabetes, respiratory disorders | 6-Shogaol | (Mao et al., 2019) |
